# Care provision and social participation among older adults in Europe: longitudinal evidence from the Survey of Health, Ageing and Retirement in Europe and the English Longitudinal Study of Ageing

**DOI:** 10.1007/s10433-025-00856-y

**Published:** 2025-06-01

**Authors:** Pamela Almeida-Meza, Giorgio Di Gessa, Rebecca Lacey, Anne McMunn, Baowen Xue

**Affiliations:** 1 Department of Epidemiology and Public Health, University College London, London, UK; 2 Population Health Research Institute, City St George’s, University of London, London, UK

**Keywords:** Care, Caregiving, Social participation, Volunteering, Older age

## Abstract

Providing care to family members and friends in older age is common, but it can impact the carers’ time and energy for social participation. This study explores the relationship between care and social participation in 16 European countries, considering factors like care status, care frequency, relationship to the care recipient, gender, socioeconomic status, and country care regimes. The study utilised pooled data from the Survey of Health Ageing and Retirement in Europe and the English Longitudinal Study of Ageing. Multiple regression models assessed the association between care status (non-carer, carer, and former carer) and social participation measured through volunteering frequency and group membership. The models adjusted for various demographic and socioeconomic covariates. The study found that carers, especially those doing so more frequently (daily and weekly care), were more likely to volunteer and belong to groups compared to non-carers. Furthermore, compared to non-carers individuals caring for their partner, parent, or non-relatives were more likely to engage in social participation. The association between care and social participation appeared stronger for carers in countries with supportive care regimes. The findings support the idea that care and social participation are complementary activities, where engagement in one represents an avenue for greater participation in the other.

**Supplementary Information:**

The online version contains supplementary material available at 10.1007/s10433-025-00856-y.

## Introduction

The increase in life expectancy over the past decades has been accompanied by an escalation in the number of years living with debilitating health conditions requiring long-term care (Colombo et al. 2011). Current European estimates suggest that around 27% of people over the age of 65 report severe difficulties in personal care or household activities (European Commission 2021). Consequently, many welfare systems across European countries rely to some degree on care to support the long-term needs of older people (Verbakel 2018). Care—also referred to in the literature as unpaid, informal, or family care or caregiving—is generally defined as non-professional (often) unpaid support given to individuals with a chronic illness or disability, with whom the carer has a social relationship (Tur-Sinai et al. 2020). In Europe, it has been estimated that 13 to 22% of adults over the age of 50 are carers and as the prevalence of the older population continues to grow, pressure on carers is expected to increase accordingly (Ribeiro et al. 2022; Tur-Sinai et al. 2020).

Social participation refers to formal and informal activities often held outside of the home, such as volunteering and engagement in community groups, which enable interaction with others in the community (Levasseur et al. 2010). Older age social participation is often motivated by the opportunity to take part in meaningful activities and has been associated with an overall reduction in morbidity and mortality (Barbosa et al. 2022; Douglas et al. 2017; Holmes and Joseph 2011; Lakomý, 2021; Pohl et al. 2022). However, social participation can be affected by care responsibilities, and this association can be further conflated by social norms, resources, and country-specific welfare policies (Choi et al. 2007; Lakomý, 2021; Quashie et al. 2022). Role theory provides a useful framework for research investigating role shifts and the clustering of time commitments in older age such as the association between care and social participation.

## Background

### Role theory: role extension and role overload

Studies finding a positive association between care and social participation in older age support the proposition that these activities might be linked by motivation and opportunity (role extension hypothesis) (Burr et al. 2005; Choi et al. 2007; Hank and Stuck 2008; Wilson and Musick 1997). For example, carers might consider volunteering as an extension of their helping role and have more opportunities for social participation due to increased contact with social and political organisations (Burr et al. 2007; Choi et al. 2007). Supporting this, previous evidence has suggested that older adults who provide more hours of care also report frequent volunteering (Burr et al. 2005). Similarly, group activities—which can include engagement in sports, belonging to organisations and clubs, and participation in leisure activities—can also represent a respite from care commitments, compensating for the potential emotional burden and stress of the role (Crittenden et al. 2022; Vlachantoni et al. 2020). Research suggests that amongst carers, those who engage in social participation report better mental health, higher life satisfaction, and more positive appraisals about the care role than carers who do not engage in social participation (Barbosa et al. 2022; Liu et al. 2023).

However, care may also be in competition with social participation since its obligatory nature can limit the ability to engage in other activities, particularly for older individuals (role overload hypothesis) (Barbosa et al. 2022; Burr et al. 2007; Li et al. 2023; Liu et al. 2021; Patterson et al. 2023). In line with this, ageing theories have argued that the number of activities individuals engage in decreases with age in response to a normative reduction in biological, mental, and social reserves (Baltes and Baltes 1990; Burr et al. 2007; Pinto and Neri 2017). Furthermore, restrictions to social participation due to caring responsibilities have been found to be associated with reduced social interaction, worse health outcomes, and reduced ability to continue care (Li et al. 2023; Liu et al. 2021; Mausbach et al. 2011; Vlachantoni et al. 2020; Wang et al. 2022). Thus, loneliness and isolation are frequently reported among older carers (Greenwood et al. 2019; Vasileiou et al. 2017).

Additionally, care characteristics might also shape the carer’s experiences of role extension or role overload. In terms of intensity or frequency of care, research has suggested that care inside the household and extended hours of care are associated with worse outcomes, including reduced social participation (Bom and Stöckel 2021; Fan et al. 2022; Kaschowitz and Brandt 2017; Patterson et al. 2023; Pohl et al. 2022). Furthermore, the literature on the relationship to the care recipient has consistently found that spousal carers tend to show worse outcomes, including reduced social participation (Barbosa et al. 2022; Li et al. 2023; Liu et al. 2021).

### Individual and contextual modifiers of care and social participation

Other dimensions influencing the link between care and social participation concern individual and contextual factors. Previous evidence on inequalities in care has found that lower socioeconomic resources are associated with a higher incidence of older adults’ care provision within the household, and that approximately two thirds of care is performed by women (Hoffmann and Rodrigues 2010; Hong Ong et al. 2024; Quashie et al. 2022). Furthermore, a recent systematic review investigating care and various health outcomes revealed that female carers were more negatively affected by the care tasks than male carers (Bom et al. 2019). At a contextual level, the division of care responsibilities between the state and the family depends on the country’s care regimes which vary by the extent to which they unburden families from care responsibilities (Defamilialism) or foster dependencies among family members (Familialism) (Brandt 2013; Kaschowitz and Brandt 2017; Zigante 2018). European studies investigating how socioeconomic resources at the national level influence care provision suggest that increased public transfers and social services reduce the burden and intensity of caregiving among older adults (Brandt 2013; Quashie et al. 2022; Verbakel 2018). Thus, care regimes influence the extent to which individuals take on additional care responsibilities (care extension) or are overwhelmed by care demands (care overload) across different countries (Bom and Stöckel 2021).

While previous research has found a relationship between care provision and social participation, the direction of this association remains unclear. Some studies suggest care provision promotes social participation (Burr et al. 2005; Hank and Stuck 2008) while others indicate that care restricts these activities due to time constraints and care burden (Liu et al. 2021; Patterson et al. 2023). Furthermore, there is limited research investigating how this association varies by care characteristics, such as frequency and relationship to the care recipient, and by individual and contextual factors such as gender, wealth and country care regimes.

This study contributes to the literature by addressing these gaps, using European data to explore how care provision and social participation are linked. The study explores two research questions: (1) whether care status (non-carer, carer, and former carer) and care characteristics, such as frequency and relationship to the care recipient, are associated with social participation; and (2) how gender, household wealth, and care regimes moderate the association between care provision and social participation. The following hypotheses are proposed. Hypothesis 1: compared to non-carers, older adults who provide care will report lower levels of social participation while former carers will report social participation levels similar to non-carers. Hypothesis 2: the negative association between care provision (carers vs non-carers) and social participation will be stronger for those providing frequent care and those caring for their partner. Hypothesis 3: the association between care status and social participation will be moderated by gender, household wealth, and care regimes. Specifically, men, those with higher wealth, and living in Defamilial regimes will report higher levels of social participation than women, those with lower wealth, and living in countries with less supportive regimes.

## Methods

### Study population

The Survey of Health, Ageing, and Retirement in Europe (SHARE) is a biennially longitudinal household survey that interviews individuals aged 50 years or older (and their partner). SHARE started in 2004, and 28 different countries have taken part ever since. This analysis used data from waves 1 (2004/05), 2 (2006/07), 4 (2011/12), 5 (2013), 6 (2015), and 8 (2019/20). SHARE waves 3 (2008/09) and 7 (2017) were excluded from the study because they focused on the participants’ life histories (see Supplementary Figs. 1–2 for timeline of study variables). For this study, the longest follow-up was from wave 1 to wave 8 and the shortest follow-up was from wave 5 to wave 8.

The English Longitudinal Study of Ageing (ELSA) is a representative longitudinal panel study designed to collect data from individuals aged 50 years and older from England. Data collection started in 2002 and is conducted biennially with refreshment samples joining the study at various stages. To be temporally consistent with SHARE, this analysis used data from waves 2 (2004/05), 3 (2006/07), 4 (2008/09), 5 (2010/11), 6 (2012/13), 7 (2014/15) and 9 (2018/19). For this study, the longest follow-up was from wave 2 to wave 9, and the shortest follow-up was from wave 6 to wave 9.

The analytical sample for the present study was restricted to participants aged 50 or above, living in a European country, and who were non-carers at their baseline assessment (i.e. first time participating in the survey). The pooled dataset was comprised of 14,809 participants for the group membership outcome and 15,555 for the volunteering frequency outcome. Missing data ranged between 0.02 and 17%; see Supplementary Fig. 3 for participant flowchart. All analyses were carried out using complete cases.

### Predictors

#### Care status

Participants were included in this study if, at their baseline assessment (ranging from 2004 to 2013, depending on when participants joined the study), they were non-carers. Care status was then measured dynamically by updating participants’ care status at each follow-up wave up to 2015 (see Supplementary Fig. 4 for care variable patterns). This approach captured individuals who did not transition to care (non-carer), transitions into care provision (carer), as well as cases where participants became carers and subsequently transitioned back to non-carers by 2015 (former carer). Therefore, the care status variable included three categories: (i) non-carer, (ii) carer, and (iii) former carer. In SHARE a positive answer to either of the questions “In the last twelve months have you personally given personal care or practical household help to a family member from outside the household, a friend or neighbour?” or “Is there someone living in this household whom you have helped regularly during the last twelve months with personal care?” was used to indicate care provision. Similarly, in ELSA, a positive answer to the question “Did you look after (i.e. active provision of care) anyone in the past week?” and choosing the option “cared for someone” after the question “Did you do any of these activities during the last month?” were used to indicate care provision.

#### Frequency of care

Frequency of care was assessed in 2015 using information on care frequency (daily, weekly, or monthly) and location of care (inside or outside the household) from each survey. In SHARE, the frequency of care was assessed using the question “How often do you give help?” which enquires on daily, weekly, monthly, or less frequent care to up to three individuals. This was restricted to personal care only. Location of care was assessed with responses to either of the two care questions used to assess care status (see paragraph above) which enquire about care outside or inside the household. In ELSA care frequency was derived using information from the two care questions (see paragraph above) which ask about care in the past week and past month. Additionally, participants were asked “How many hours in the past week did you do this?” with those answering more than 10 h being categorised as daily carers, and those reporting 10 or less hours being categorised as weekly carers (Di Gessa & Deindl, 2024). Location of care was assessed in the response to the question “does the person/any of the people you care for live with you?” (yes/no). This process resulted in a harmonised variable with five categories of frequency of care: (i) non-carer, (ii) cared monthly, (iii) cared outside the household weekly, (iv) cared outside the household daily, and (v) cared inside the household daily*.*

#### Relationship to care recipient

The relationship to care recipient was measured in 2015 using six indicator variables including (i) spouse/partner, (ii) child, (iii) parent, (iv) parent-in-law, (v) other relative, and (vi) non-relative. SHARE excluded caring for grandchildren and therefore this restriction was transferred to ELSA.

#### Care regimes

Based on the three-factor solution from Van Damme and colleagues in this issue (van Damme et al. 2024), the 16 European countries were grouped as follows: (i) Defamilialism group (DEF—high support) which included Denmark and Sweden; (ii) Moderate Defamilialism/Supported Familialism group (MD/SF—medium support) which included England, Austria, Germany, Spain, France, Switzerland, Belgium, and Luxembourg; and (iii) Familialism group (FbD—low support) which included Italy, Greece, Czech Republic, Poland, Slovenia and Estonia.

### Outcome

Outcome data, volunteering frequency and group membership, were measured at two time points: 2015 (short-term follow-up), and 2018/19 (long-term follow-up).

#### Volunteering

In SHARE frequency of voluntary work was derived from answers (ranging from almost every day to every few months) to the question “how often have you done voluntary/charity work in the last 12 months”, whereas in ELSA it was derived from answers (ranging from twice a month to once or twice a year) to the question “how often do you do any voluntary work?”. The resulting harmonised variable had four levels of frequency: (i) twice a month or more, (ii) almost every month, (iii) every few months or less often, and (iv) no volunteering.

#### Group membership

Amongst the social participation variables included in ELSA and SHARE three group membership variables could be compared across both studies. The variables included (i) attending an educational or training course; (ii) gone to a sport, social, or other kind of club; and (iii) taken part in a political or environmental organisation. A count variable (ranging from 0 to 3) was created to reflect the number of social groups participants were involved in. However, due to the low number of participants reporting belonging to three groups (< 2%, see Table 1), for the main analysis the variable was dichotomised representing (i) no group membership, or (ii) membership of 1 to 3 groups.

**Table 1:** Sample characteristics by care status for participants present in volunteering frequency and group membership samples (*N* = 14,806)

Variables	Care status N % or Mean (SD)					
	Non-carer		Carer		Former carer	
	11,337	76.57	1299	8.77	2170	14.66
*Baseline age*	63.27	(8.55)	59.96	(7.30)	61.81	(7.78)
*Baseline group membership*						
No membership	7343	64.77	725	55.81	1393	64.19
1 group mentioned	3107	27.41	411	31.64	584	26.91
2 groups mentioned	774	6.83	142	10.93	165	7.60
3 groups mentioned	113	1.00	21	1.62	28	1.29
*Baseline voluntary work*						
No volunteering or charity work	9586	84.55	967	74.44	1711	78.85
Every few months or less often	420	3.70	96	7.39	136	6.27
Almost every month	425	3.75	50	3.85	61	2.81
Twice a month or more	906	7.99	186	14.32	262	12.07
*Care frequency*						
Daily care in household	–	–	493	37.95	–	–
Daily care outside household	–	–	291	22.40	–	–
Weekly care outside household	–	–	426	32.79	–	–
Monthly care	–	–	89	6.85	–	–
*Care relationship*						
Looked after partner/spouse	–	–	386	29.72	–	–
Looked after child	–	–	238	18.32	–	–
Looked after parent	–	–	365	28.10	–	–
Looked after parents-in-law	–	–	113	8.70	–	–
Looked after other relatives	–	–	138	10.62	–	–
Looked after non-relatives	–	–	301	23.17	–	–
*Country care regime*						
Defamilialism	1022	9.01	93	7.16	218	10.05
Moderate Defamilialism/Supported Familialism	6129	54.06	838	64.51	1237	57.00
Defamilialism-by-default	4186	36.92	368	28.33	715	32.95
*Current work*						
Retired	5806	51.21	500	38.49	991	45.67
Employed or self-employed full-time	3711	32.73	590	45.42	793	36.54
Employed or self-employed part-time	140	1.23	30	2.31	37	1.71
Unemployed	343	3.03	28	2.16	64	2.95
Permanently sick or disabled	408	3.60	42	3.23	65	3.00
Homemaker	929	8.19	109	8.39	220	10.14
*Education level*						
Less than upper secondary education	4160	36.69	390	30.02	779	35.90
Upper secondary and vocational training	4823	42.54	601	46.27	899	41.43
Tertiary education	2354	20.76	308	23.71	492	22.67
*Frequency voluntary work (2015)*						
No volunteering or charity work	9537	84.12	905	69.67	1699	78.29
Every few months or less often	397	3.50	53	4.08	98	4.52
Almost every month	433	3.82	86	6.62	103	4.75
Twice a month or more	970	8.56	255	19.63	270	12.44
*Frequency voluntary work (2019)*						
No volunteering or charity work	9562	84.34	918	70.67	1730	79.72
Every few months or less often	412	3.63	72	5.54	76	3.50
Almost every month	405	3.57	58	4.46	76	3.50
Twice a month or more	958	8.45	251	19.32	288	13.27
*Group membership (2015)*						
No membership	7312	64.50	685	52.73	1312	60.46
1 group mentioned	3093	27.28	437	33.64	655	30.18
2 groups mentioned	790	6.97	151	11.62	166	7.65
3 groups mentioned	142	1.25	26	2.00	37	1.71
*Group membership (2019)*						
No membership	7473	65.92	724	55.74	1383	63.73
1 group mentioned	3047	26.88	428	32.95	602	27.74
2 groups mentioned	719	6.34	124	9.55	160	7.37
3 groups mentioned	98	0.86	23	1.77	25	1.15
*Household wealth*						
1 (lowest quintile)	2787	24.58	338	26.02	531	24.47
2	1565	13.80	176	13.55	286	13.18
3	2098	18.51	233	17.94	443	20.41
4	2298	20.27	273	21.02	430	19.82
5 (highest quintile)	2589	22.84	279	21.48	480	22.12
*Limiting long-term illness*						
No illness	5977	52.72	707	54.43	1120	51.61
Not limiting illness	1824	16.09	233	17.94	417	19.22
Limiting illness	3536	31.19	359	27.64	633	29.17
*Live with partner*						
Yes	8800	77.62	1105	85.07	1602	73.82
No	2537	22.38	194	14.93	568	26.18
*Mental health caseness*						
No	8288	73.11	852	65.59	1498	69.03
Yes	3049	26.89	447	34.41	672	30.97
*Gender*						
Men	5174	45.64	442	34.03	825	38.02
Women	6163	54.36	857	65.97	1345	61.98

### Covariates

Covariates were only measured at participants’ baseline assessment (ranging from 2004 to 2013) when they were non-carers. The covariates included in this study were baseline volunteering frequency, baseline group membership, country care regime (DEF, MD/SF, FbD), age (continuous variable, range 50 to 92), gender (women/men), living with their partner (yes/no), education (less than upper secondary education, upper secondary and vocational training, tertiary education), current employment status (retired, employed or self-employed full-time, employed or self-employed part-time, unemployed, permanently sick or disabled, homemaker), equivalised household non-housing wealth (quintiles), longstanding limiting illness (no illness, not limiting long-term illness, limiting long-term illness), and mental health caseness (yes/no) measured using the Centre for Epidemiological Studies Depression 8 (CES-D 8) in ELSA and EURO-D in SHARE, and identified as scores of 3 or more in CES-D scale of 4 or more in the EURO-D scale (Castro-Costa et al. 2008; White et al. 2015).

## Analysis

Descriptive characteristics of the study sample and care characteristics were explored stratified by care status. Due to the differing sample sizes for volunteering frequency and group membership, descriptive analysis was carried out only for the participants with data for both outcomes (see Supplementary Fig. 3 for participant flowchart). To assess the association between care and social participation, multinomial logistic regressions for volunteering frequency, and logistic regressions for group membership were carried out. To facilitate interpretation, the coefficients for the multinomial logistic regressions are presented as average marginal effects (see supplementary material for incidence rate ratios). Two sets of models were run for each outcome. The first model explored the unadjusted association between care and social participation and the second model adjusted for all covariates, including the potential moderators. Finally, to test for differences by country care regime, household wealth, and gender, two-way interaction terms (care status x each moderator) were included in the association between care and social participation. Three-way interactions (care status x gender or wealth x regime) were also included to explore how gender or wealth differences differed by care regime. Wald tests were carried out to test the findings from the interactions.

Two sensitivity analyses were carried out. The sample for the main analysis includes participants with outcome data in both, 2015 and 2019. However, this might increase the risk of attrition bias, particularly since SHARE wave 8 data collection was affected by the COVID-19 pandemic. Thus, the first sensitivity analysis was carried out with the 2015 outcome (short-term follow-up) including all participants available at this stage. The second sensitivity analysis examined how care status at the long-term follow-up may influence social participation by controlling for care in 2019.

All analyses were carried out using Stata MP version 17 (StataCorp 2020).

## Results

### Descriptive statistics

The average age of the participants was 63 years and 56.50% of the sample were female. Compared to non-carers, carers appeared to be slightly younger, and females represented the largest proportion of those with care experience (i.e. carers or former carers) (see Table 1). At baseline, the sample reported low social participation with 82.83% of all participants reporting no volunteering activities and 63.90% reporting no group membership. Most participants in the sample lived in countries classified under the MD/SF care regimes. In terms of living arrangements, 77.72% of the sample reported living with their partner, with carers reporting living with their partners more often than non-carers and former carers. Furthermore, compared to non-carers, carers reported a lower proportion of mental health caseness and long-term limiting illnesses, and a higher proportion of university degrees and full-time work.

The categorisation of participants into care groups revealed that 8.77% of the sample became carers between 2004 and 2013, whereas most of the sample remained as non-carers (76.57%), and around 14.66% were former carers. Amongst carers, the majority (62.04%) provided daily or weekly care outside the household or provided care monthly. Furthermore, the relationships to care recipient most frequently mentioned were partner or spouse (29.72%) and parents (28.10%), whereas the least mentioned were other relatives (10.62%) and parents-in-law (8.70%).

In 2015, the adjusted model suggested that compared to non-carers, carers were more likely to volunteer almost every month (AME = 0.02, 95% CI 0.00–0.03) and 6 percentage points more likely to volunteer twice a month or more often (AME = 0.06, 95% CI 0.04–0.07) (see Table 2); only the association between care and volunteering twice a month or more often remained in 2019. Furthermore, the 2015 and 2019 analyses suggested that former carers were more likely to volunteer twice a month or more than non-carers (2015 AME = 0.01, 95% CI 0.00–0.03; 2019 AME = 0.03, 95% CI 0.001–0.04). See Supplementary Table 1 for covariates’ coefficients.

**Table 2:** Care status, care frequency, relationship and volunteering frequency, 2015 and 2019 results (N = 15,555)

	2015						2019					
	Unadjusted			Adjusted			Unadjusted			Adjusted		
	AME	95% CI		AME	95% CI		AME	95% CI		AME	95% CI	
Care status												
Non-care (Reference)	–	–	–	–	–	–	–	–	–	–	–	–
Carer												
No volunteering	− 0.14**	− 0.16	− 0.11	− 0.07**	− 0.09	− 0.05	− 0.13**	− 0.15	− 0.10	− 0.06**	− 0.07	− 0.04
Every few months or less often	0.01	− 0.01	0.02	0.00	− 0.01	0.01	0.02*	0.00	0.03	0.01	− 0.00	0.02
Almost every month	0.03**	0.01	0.04	0.02*	0.00	0.03	0.01	− 0.00	0.02	− 0.00	− 0.01	0.01
Twice a month or more	0.11**	0.08	0.13	0.06**	0.04	0.07	0.10**	0.08	0.12	0.05**	0.03	0.07
Former carer												
No volunteering	− 0.06**	− 0.08	− 0.04	− 0.03**	− 0.04	− 0.01	− 0.05**	− 0.07	− 0.03	− 0.01	− 0.03	0.00
Every few months or less often	0.01*	0.00	0.02	0.01	− 0.00	0.02	0.00	− 0.01	0.01	− 0.00	− 0.01	0.00
Almost every month	0.01	− 0.00	0.02	0.00	− 0.00	0.01	0.00	− 0.01	0.01	− 0.01	− 0.01	0.00
Twice a month or more	0.04**	0.03	0.05	0.01*	0.00	0.03	0.05**	0.04	0.07	0.03**	0.01	0.04
Care frequency												
Non-care (Reference)	–	–	–	–	–	–	–	–	–	–	–	–
Former carer												
No volunteering	− 0.06**	− 0.08	− 0.04	− 0.03**	− 0.04	− 0.01	− 0.05**	− 0.07	− 0.03	− 0.01	− 0.03	0.00
Every few months or less often	0.01*	0.00	0.02	0.01	0.00	0.02	0.00	− 0.01	0.01	0.00	− 0.01	0.00
Almost every month	0.01	− 0.00	0.02	0.00	0.00	0.01	0.00	− 0.01	0.01	− 0.01	− 0.01	0.00
Twice a month or more	0.04**	0.03	0.05	0.01*	0.00	0.03	0.05**	0.04	0.07	0.03**	0.01	0.04
Daily care in household												
No volunteering	− 0.09**	− 0.13	− 0.06	− 0.06**	− 0.09	− 0.03	− 0.07**	− 0.11	− 0.04	− 0.04*	− 0.07	− 0.01
Every few months or less often	0.01	− 0.01	0.02	0.00	− 0.01	0.02	0.01	− 0.00	0.03	0.01	− 0.01	0.03
Almost every month	0.03*	0.00	0.05	0.02*	0.00	0.04	0.00	− 0.02	0.01	− 0.01	− 0.02	0.01
Twice a month or more	0.06**	0.03	0.09	0.03*	0.01	0.06	0.06**	0.03	0.09	0.04**	0.01	0.06
Daily care outside household												
No volunteering	− 0.11**	− 0.16	− 0.06	− 0.04*	− 0.08	− 0.01	− 0.13**	− 0.19	− 0.08	− 0.05*	− 0.09	− 0.01
Every few months or less often	0.00	− 0.02	0.03	− 0.01	− 0.02	0.01	0.02	− 0.01	0.04	0.00	− 0.02	0.02
Almost every month	0.00	− 0.02	0.02	− 0.01	− 0.03	0.01	0.01	− 0.01	0.03	0.00	− 0.02	0.02
Twice a month or more	0.11**	0.07	0.16	0.06**	0.03	0.09	0.11**	0.07	0.15	0.05**	0.02	0.08
Weekly care outside household												
No volunteering	− 0.20**	− 0.24	− 0.15	− 0.09**	− 0.12	− 0.06	− 0.20**	− 0.24	− 0.15	− 0.08**	− 0.11	− 0.05
Every few months or less often	0.01	− 0.01	0.02	0.00	− 0.02	0.01	0.02	− 0.00	0.04	0.01	− 0.01	0.03
Almost every month	0.03*	0.00	0.05	0.02	− 0.00	0.04	0.00	− 0.01	0.02	− 0.01	− 0.02	0.01
Twice a month or more	0.16**	0.12	0.20	0.08**	0.05	0.10	0.17**	0.13	0.21	0.08**	0.05	0.10
Monthly care												
No volunteering	− 0.19**	− 0.29	− 0.09	− 0.13**	− 0.21	− 0.05	− 0.08	− 0.17	0.01	− 0.02	− 0.08	0.05
Every few months or less often	0.01	− 0.03	0.05	0.00	− 0.04	0.04	0.00	− 0.04	0.03	− 0.01	− 0.04	0.02
Almost every month	0.11**	0.03	0.18	0.08*	0.02	0.14	0.08*	0.01	0.14	0.05	− 0.00	0.10
Twice a month or more	0.07	− 0.00	0.15	0.04	− 0.02	0.10	0.01	− 0.05	0.07	− 0.02	− 0.07	0.03
Relationship												
Non-care (Reference)	–	–	–	–	–	–	–	–	–	–	–	–
Former carer												
No volunteering	− 0.06**	− 0.08	− 0.04	− 0.03**	− 0.04	− 0.01	− 0.05**	− 0.07	− 0.03	− 0.01	− 0.03	0.00
Every few months or less often	0.01*	0.00	0.02	0.01	0.00	0.02	0.00	− 0.01	0.01	0.00	− 0.01	0.00
Almost every month	0.01	− 0.00	0.02	0.00	0.00	0.01	0.00	− 0.01	0.01	− 0.01	− 0.01	0.00
Twice a month or more	0.04**	0.03	0.05	0.01*	0.00	0.03	0.05**	0.04	0.07	0.03**	0.01	0.04
Care for partner												
No volunteering	− 0.08**	− 0.12	− 0.04	− 0.05*	− 0.08	− 0.01	− 0.09**	− 0.13	− 0.05	− 0.06**	− 0.09	− 0.02
Every few months or less often	0.00	− 0.02	0.02	0.00	− 0.02	0.02	0.00	− 0.01	0.02	0.00	− 0.02	0.02
Almost every month	0.02	0.00	0.04	0.01	− 0.01	0.03	0.00	− 0.02	0.02	0.00	− 0.02	0.01
Twice a month or more	0.06**	0.03	0.10	0.04*	0.01	0.07	0.08**	0.05	0.12	0.06**	0.03	0.09
Care for child												
No volunteering	− 0.05	− 0.10	0.00	− 0.04	− 0.08	0.01	− 0.07*	− 0.13	− 0.02	− 0.06*	− 0.10	− 0.01
Every few months or less often	− 0.01	− 0.03	0.01	− 0.01	− 0.03	0.01	0.05*	0.01	0.08	0.05*	0.01	0.08
Almost every month	0.03	0.00	0.06	0.02	− 0.00	0.05	− 0.01	− 0.03	0.01	− 0.01	− 0.03	0.01
Twice a month or more	0.03	− 0.01	0.07	0.02	− 0.01	0.06	0.03	− 0.01	0.07	0.02	− 0.01	0.06
Care for parent												
No volunteering	− 0.12**	− 0.16	− 0.07	− 0.06**	− 0.09	− 0.02	− 0.11**	− 0.16	− 0.07	− 0.03	− 0.06	0.00
Every few months or less often	0.02	0.00	0.04	0.01	− 0.01	0.03	0.03*	0.01	0.06	0.02	− 0.00	0.04
Almost every month	0.03*	0.01	0.06	0.02	− 0.00	0.05	0.02	− 0.01	0.04	0.00	− 0.01	0.02
Twice a month or more	0.06**	0.03	0.10	0.03	− 0.00	0.05	0.06**	0.02	0.10	0.01	− 0.02	0.03
Care for parent-in-law												
No volunteering	− 0.14**	− 0.22	− 0.06	− 0.07*	− 0.14	− 0.01	− 0.12*	− 0.20	− 0.04	− 0.03	− 0.09	0.02
Every few months or less often	0.00	− 0.03	0.03	− 0.01	− 0.04	0.01	0.02	− 0.02	0.07	0.01	− 0.03	0.04
Almost every month	0.03	− 0.02	0.08	0.02	− 0.02	0.06	0.03	− 0.01	0.08	0.02	− 0.02	0.05
Twice a month or more	0.11**	0.04	0.18	0.07*	0.01	0.12	0.06	0.00	0.12	0.01	− 0.03	0.05
Care for other relative												
No volunteering	− 0.08*	− 0.15	− 0.01	− 0.03	− 0.08	0.03	− 0.04	− 0.11	0.02	0.01	− 0.04	0.06
Every few months or less often	0.01	− 0.03	0.04	0.00	− 0.03	0.03	− 0.01	− 0.04	0.02	− 0.02	− 0.04	0.01
Almost every month	0.02	− 0.02	0.06	0.01	− 0.02	0.04	0.01	− 0.03	0.04	0.00	− 0.03	0.03
Twice a month or more	0.05	0.00	0.11	0.02	− 0.03	0.06	0.05	− 0.01	0.10	0.01	− 0.03	0.05
Care for non-relative												
No volunteering	− 0.32**	− 0.38	− 0.27	− 0.20**	− 0.25	− 0.16	− 0.22**	− 0.28	− 0.17	− 0.11**	− 0.16	− 0.07
Every few months or less often	0.02	− 0.01	0.04	0.01	− 0.01	0.04	0.03	− 0.00	0.05	0.02	− 0.01	0.04
Almost every month	0.06**	0.03	0.10	0.05**	0.02	0.08	0.01	− 0.01	0.04	0.00	− 0.02	0.02
Twice a month or more	0.24**	0.19	0.29	0.14**	0.10	0.18	0.18**	0.13	0.23	0.10**	0.06	0.13

Adjusted model controls for outcome at baseline, care regimes, age, gender, living with partner, education, employment status, household wealth, longstanding limiting illness, and mental health symptoms

*p-value < 0.05; **p-value < 0.001

In terms of care frequency, the 2015 adjusted results suggested that compared to non-carers, individuals providing care daily in the household (AME = 0.02, 95% CI 0.00–0.04), and those doing so monthly (AME = 0.08, 95% CI 0.02–0.14) were more likely to volunteer almost every month. And those providing care daily in the household (AME = 0.03, 95% CI 0.01–0.06), daily outside the household (AME = 0.06, 95% CI 0.03–0.09), and weekly outside the household (AME = 0.08, 95% CI 0.05–0.010) were more likely to volunteer twice a month or more. Except for monthly care, these results persisted by 2019.

Additionally, compared to non-carers, the 2015 adjusted models suggested that participants providing care for non-relatives (AME = 0.05, 95% CI 0.02–0.08) were more likely to volunteer almost every month (see Table 2). Similarly, participants providing care for a partner (AME = 0.04, 95% CI 0.01–0.07), parent-in-law (AME = 0.07, 95% CI 0.01–0.12), and non-relative (AME = 0.14, 95% CI 0.10–0.18) were more likely to volunteer twice a month or more. However, in the 2019 models, only those providing care for a partner or non-relatives remained more likely to volunteer frequently, while the association was no longer observed for other care groups.

The 2015 interaction analyses suggested that country care regimes moderated the association between care and volunteering frequency. However, the stratified coefficients and corresponding overlapping confidence intervals suggest a consistent pattern across care regimes (see Supplementary Fig. 5).

### Care and group membership

The 2015 and 2019 adjusted analyses suggested that carers had higher odds of belonging to a group than non-carers (2015 OR = 1.39, 95% CI 1.21–1.59; 2019 OR = 1.21, 95% CI 1.06–1.39) (see Table 3). Furthermore, in 2015 former carers (OR = 1.16, 95% CI 1.04–1.30) had higher odds of belonging to a group than non-carers, however, this association was non-significant in the 2019 analysis. See supplementary Table 5 for covariates’ coefficients.

**Table 3:** Care category, care frequency, relationship and group membership, 2015 and 2019 results (*N* = 14,809)

	2015						2019					
	Unadjusted			Adjusted			Unadjusted			Adjusted		
	OR	95% CI		OR	95% CI		OR	95% CI		OR	95% CI	
*Care status*												
Non-carer (Reference)	–	–	–	–	–	–	–	–	–	–	–	–
Carer	1.63**	1.45	1.83	1.39**	1.21	1.59	1.54**	1.37	1.73	1.21*	1.06	1.39
Former carer	1.19**	1.08	1.31	1.16*	1.04	1.30	1.10	1.00	1.21	1.02	0.91	1.14
*Care frequency*												
Non-carer (Reference)	–	–	–	–	–	–	–	–	–	–	–	–
Former carer	1.19**	1.08	1.31	1.16*	1.04	1.30	1.10	1.00	1.21	1.02	0.91	1.14
Daily in household	1.29*	1.07	1.55	1.30*	1.05	1.62	1.25*	1.04	1.50	1.23	0.99	1.52
Daily outside household	1.80**	1.43	2.28	1.53**	1.16	2.01	1.56**	1.24	1.97	1.16	0.88	1.52
Weekly outside household	1.87**	1.54	2.27	1.42**	1.13	1.78	1.79**	1.48	2.18	1.24	0.99	1.55
Monthly	2.23**	1.46	3.38	1.29	0.79	2.11	2.16**	1.42	3.29	1.20	0.74	1.94
*Relationship*												
Non-carer (Reference)	–	–	–	–	–	–	–	–	–	–	–	–
Former carer	1.19**	1.08	1.31	1.16*	1.04	1.30	1.10	1.00	1.21	1.02	0.91	1.14
Partner	1.33*	1.09	1.64	1.38*	1.09	1.77	1.14	0.93	1.41	1.13	0.89	1.44
Child	1.53**	1.19	1.99	1.74**	1.28	2.37	1.22	0.94	1.59	1.22	0.89	1.66
Parent	2.30**	1.87	2.84	1.61**	1.26	2.06	2.17**	1.76	2.67	1.32*	1.04	1.68
Parent-in-law	1.78**	1.23	2.59	1.24	0.80	1.93	1.28	0.88	1.87	0.74	0.48	1.13
Other relative	1.46*	1.04	2.05	1.46	0.99	2.17	1.22	0.86	1.72	1.07	0.72	1.58
Non-relative	1.95**	1.55	2.46	1.36*	1.04	1.77	1.95**	1.55	2.45	1.37*	1.06	1.78

Adjusted model controls for outcome at baseline, care regimes, age, gender, living with partner, education, employment status, household wealth, longstanding limiting illness, and mental health symptoms

*p-value < 0.05; **p-value < 0.001

The analysis showed an association between care frequency and group membership for individuals providing care daily in household (OR = 1.30, 95% CI 1.05–1.62), daily outside the household (OR = 1.53, 95% CI 1.16–2.01), and weekly outside the household (OR = 1.42, 95% CI 1.13–1.78). However, these associations were no longer significant in 2019. In addition, the adjusted 2015 models suggested that individuals providing care for partners (OR = 1.38, 95% CI 1.09–1.77), children (OR = 1.74, 95% CI 1.28–2.37), parents (OR = 1.61, 95% CI 1.26–2.06), and non-relatives (OR = 1.36, 95% CI 1.04–1.77) had higher odds of belonging to a group than non-carers. However, by 2019 only those caring for parents (OR = 1.32, 95% CI 1.04–1.68) and non-relatives (OR = 1.37, 95% CI 1.06–1.78) had significantly higher odds of belonging to a group. See Supplementary Table 6 for the distribution of care groups by individual group membership.

The interaction analysis suggested that the association between care and group membership (2019) was moderated by the country care regimes. The association between care and group membership appear to be stronger for carers in countries with DEF regimes, although the confidence intervals overlap between regimes (see Supplementary Fig. 6).

### Sensitivity analyses

The analysis carried out with the sample with data up to 2015, and the analysis that adjusted for care in 2019 confirmed the results of the main analysis. See Supplementary Tables 7–18.

## Discussion

This study investigated the association between care status (non-carer, carer, and former carer) and social participation over a period of five to 16 years using pooled data from 16 European countries. The findings suggested that care is associated with increased social participation, particularly volunteering frequency. This association was present for individuals providing frequent care (daily or weekly vs monthly) and predominantly for those doing so weekly outside the household. The findings align with broader research supporting role extension theory by suggesting that care provision is not necessarily associated with worse social outcomes and might even lead to increased activity engagement (Larkin et al. 2019; Vlachantoni et al. 2020). In line with this, international research suggests that the impact of care provision depend on the burden of the care responsibilities, with low intensity linked with emotional benefits, and high intensity associated with poorer health outcomes and reduced social participation (Bom and Stöckel 2021; Fan et al. 2022; Kaschowitz and Brandt 2017).

The positive association between care and volunteering has been reported by other studies (Burr et al. 2005; Hank and Stuck 2008). For instance, a recent investigation using data from the Health and Retirement study found that participants who volunteered were more likely to help their relatives and friends (Han et al. 2023). This provides evidence for the idea that care and volunteering are complementary helping activities and introduces the possibility of reverse causality whereby providing care and social participation foster further helping behaviours. In line with this, survey data from the USA has suggested that not only are carers more likely to volunteer than non-carers but were also more likely to be invited to volunteer (Burr et al. 2005). Additionally, the present study provided evidence for the stability and continuity of social participation over time. The findings suggested that care is associated with social participation, independent of baseline social participation or follow-up care status. And consistent with previous literature (Burr et al. 2005; Choi et al. 2007; Di Gessa and Grundy 2017; Vangen et al. 2021), the study found that participants’ baseline social participation—which preceded the transition to care—was the strongest predictor of social participation in the short- and long-term.

The present study adds to the limited research on the association between care and group membership and suggests that carers were more likely to belong to a group than non-carers—although this was only observed in the short-term follow-up. This mirrors recent findings from the National Child Development Study which suggested that adult cares are more likely to engage in social participation than their non-carer counterparts (Vlachantoni et al. 2020). Other studies have found that individuals providing more time caring (e.g. providing help with health care), show a decline in valued and community-based activities (Li et al. 2023; Patterson et al. 2023; Wolff et al. 2016), but that a sub-set of these cares (e.g. non-spouse carers) sustain or increase their social participation (Li et al. 2023; Patterson et al. 2023). On one hand, these findings call for further research to understand these dynamics and long-term effects of care on group membership. And on the other hand, the findings suggest that group membership and volunteering are distinct forms of social participation. While volunteering may serve as an extension of the helping role, group membership might reflect personal and recreational engagement. Thus, it is possible that for some individuals, group membership may be more susceptible to role overload due to time constraints and shifting priorities.

Providing care for all care recipient relationships (i.e. partner, child, parent, parent-in-law, and non-relatives), except for other relatives, were associated with increased social participation in the short-term, but that only carers for partners, parents, and non-relatives maintained the association in the long term. Previous evidence for the health and social effects of caring for different individuals are mixed (Choi et al. 2007; Li et al. 2023; Li and Lee 2020). However, one study has highlighted the idea that independently of the relationship to the care recipient, those who had no choice in becoming carers experienced worse mental health outcomes (Li et al. 2023). Therefore, choosing to become a carer might be associated with increased social participation through role extension, while assuming a caring role involuntarily might lead to role overload, increasing the risk of psychological distress and social withdrawal.

The generosity of a country’s care regime has also been found to shape the opportunities for social participation among carers. DEF regimes reduce the need for family members to provide long hours of care, particularly within the household, alleviating the care burden and allowing carers to engage in other activities (Lakomý, 2021; Quashie et al. 2022). Consistent with this, the study found some evidence supporting that carers in DEF regimes show more social participation for carers than regimes providing less support.

Finally, the findings suggest that former carers engage in more social participation than non-carers. There is little evidence on the experience of this population, with existing studies focusing on the effects of the loss of the care recipient, the legacies of caring, and post-caring support services (Cavaye and Watts 2018; Mora-Lopez et al. 2022). Therefore, further research should aim to understand post-caring experiences, including factors influencing social participation and coping mechanisms.

The strengths of this study stem from the use of longitudinal data sourced from well-established panel studies. The study benefits from a large sample size and an extended observation period, facilitating the assessment of care status and its association with social participation at two distinct time points while addressing the issue of reverse causality. Moreover, the study incorporates multinational comparisons, enhancing the external validity of its findings.

However, harmonising ELSA and SHARE introduced limitations, as differences in data collection methods, variable definitions, and measurement instruments may lead to inconsistencies. For example, the differing periods for assessing care activities and social participation between the ELSA and SHARE introduces some inconsistencies in the predictor and outcome variables. Other limitations relate to the self-report nature of the variables, attrition, and the four-year time gap between waves 2 and 4 of SHARE due to the inclusion of the life history questionnaire in wave 3. In ELSA from waves 2 to 5, data on past-week care were only available for those who reported care in the past month, whereas from wave 6 the two questions were asked independently. This might have led to under-identification of carers in earlier waves. Additionally, since care status was assessed through the follow-up period, but covariates were only measured at baseline, the analyses assume that baseline characteristics remain relatively stable. Future research should explore how changes in health status, employment, and socioeconomic position may impact the association between care and social participation.

## Conclusion

The findings suggest that care provision is associated with increased social participation, particularly for volunteering and among individuals providing more frequent care (daily or weekly vs monthly). These findings contribute to the research supporting role extension theory, suggesting that care provision can foster social participation. Furthermore, the study reinforces the idea that care and volunteering are reciprocally related, where individuals who are already inclined to help others may be more likely to care and volunteer. The findings also underscore how volunteering and social participation might be distinct forms of social participation and thus, might be affected differently by care provision. Furthermore, the study highlights the importance of considering contextual factors in understanding carers’ social participation outcomes and further supports the relevance of the availability and accessibility of resources and opportunities for the quality life of older adults providing care.

## Supplementary Information

Below is the link to the electronic supplementary material.

Supplementary file1 (DOCX 706 KB)

## Data Availability

Researchers can download ELSA data from all waves, from the UK Data Service. For more information, please visit https://www.elsa-project.ac.uk/accessing-elsa-data. All waves of SHARE data can be accessed via https://share-eric.eu/data/data-access.
