# Supplementary material for: Care provision and social participation among older adults in Europe: longitudinal evidence from the Survey of Health, Ageing and Retirement in Europe and the English Longitudinal Study of Ageing
Source: Eur J Ageing. 2025 Jun 1;22(1):24. doi: 10.1007/s10433-025-00856-y (PMC12127247; doi:10.1007/s10433-025-00856-y)
Supplement: Supplementary file 1 — Supplementary file1 (DOCX 706 KB) [file 10433_2025_856_MOESM1_ESM.docx]

**Title:**

Care provision and social participation among older adults in Europe: Longitudinal Evidence from the Survey of Health, Ageing and Retirement in Europe and the English Longitudinal Study of Ageing

**Journal:**

European Journal of Ageing

**Author information:**

Pamela Almeida-Meza (corresponding author)

University College London, Department of Epidemiology & Public Health, London, United Kingdom

p.meza.17@ucl.ac.uk

Giorgio Di Gessa

Rebecca Lacey

Anne McMunn

Baowen Xue

**Supplementary Table 1.** Care status and volunteering frequency adjusted models, 2015 and 2019 results (N=15,555)

|  | **2015** | | | | **2019** | | | |
| --- | --- | --- | --- | --- | --- | --- | --- | --- |
|  | AME | p-value | 95% CI | | AME | p-value | 95% CI | |
| **Care status** |  |  |  |  |  |  |  |  |
| Non-carer (reference) | - | - | - | - | - | - | - | - |
| Carer |  |  |  |  |  |  |  |  |
| No volunteering | -0.07 | 0.00 | -0.09 | -0.05 | -0.06 | 0.00 | -0.07 | -0.04 |
| Every few months or less often | -0.00 | 0.79 | -0.01 | 0.01 | 0.01 | 0.23 | -0.00 | 0.02 |
| Almost every month | 0.02 | 0.01 | 0.00 | 0.03 | -0.00 | 0.73 | -0.01 | 0.01 |
| Twice a month or more | 0.06 | 0.00 | 0.04 | 0.07 | 0.05 | 0.00 | 0.03 | 0.07 |
| Former carer |  |  |  |  |  |  |  |  |
| No volunteering | -0.03 | 0.00 | -0.04 | -0.01 | -0.01 | 0.05 | -0.03 | 0.00 |
| Every few months or less often | 0.01 | 0.09 | -0.00 | 0.02 | -0.00 | 0.22 | -0.01 | 0.00 |
| Almost every month | 0.00 | 0.32 | -0.00 | 0.01 | -0.01 | 0.14 | -0.01 | 0.00 |
| Twice a month or more | 0.01 | 0.02 | 0.00 | 0.03 | 0.03 | 0.00 | 0.01 | 0.04 |
|  |  |  |  |  |  |  |  |  |
| **Baseline volunteering frequency** |  |  |  |  |  |  |  |  |
| No volunteering (reference) | - | - | - | - | - | - | - | - |
| Every few months or less often |  |  |  |  |  |  |  |  |
| No volunteering | -0.20 | 0.00 | -0.24 | -0.17 | -0.17 | 0.00 | -0.20 | -0.14 |
| Every few months or less often | 0.08 | 0.00 | 0.06 | 0.10 | 0.07 | 0.00 | 0.05 | 0.09 |
| Almost every month | 0.06 | 0.00 | 0.04 | 0.08 | 0.04 | 0.00 | 0.02 | 0.06 |
| Twice a month or more | 0.07 | 0.00 | 0.05 | 0.09 | 0.07 | 0.00 | 0.04 | 0.09 |
| Almost every month |  |  |  |  |  |  |  |  |
| No volunteering | -0.33 | 0.00 | -0.37 | -0.29 | -0.31 | 0.00 | -0.35 | -0.27 |
| Every few months or less often | 0.06 | 0.00 | 0.03 | 0.08 | 0.06 | 0.00 | 0.04 | 0.09 |
| Almost every month | 0.14 | 0.00 | 0.11 | 0.17 | 0.10 | 0.00 | 0.07 | 0.13 |
| Twice a month or more | 0.13 | 0.00 | 0.10 | 0.16 | 0.15 | 0.00 | 0.12 | 0.18 |
| Twice a month or more |  |  |  |  |  |  |  |  |
| No volunteering | -0.43 | 0.00 | -0.46 | -0.40 | -0.35 | 0.00 | -0.37 | -0.32 |
| Every few months or less often | 0.01 | 0.05 | -0.00 | 0.02 | 0.02 | 0.00 | 0.01 | 0.04 |
| Almost every month | 0.07 | 0.00 | 0.05 | 0.09 | 0.06 | 0.00 | 0.04 | 0.07 |
| Twice a month or more | 0.35 | 0.00 | 0.32 | 0.38 | 0.26 | 0.00 | 0.24 | 0.29 |
|  |  |  |  |  |  |  |  |  |
| **Regime** |  |  |  |  |  |  |  |  |
| High support (reference) | - | - | - | - | - | - | - | - |
| Medium support |  |  |  |  |  |  |  |  |
| No volunteering | -0.02 | 0.04 | -0.04 | -0.00 | -0.02 | 0.12 | -0.04 | 0.00 |
| Every few months or less often | 0.00 | 0.60 | -0.01 | 0.01 | 0.01 | 0.19 | -0.00 | 0.02 |
| Almost every month | -0.00 | 0.68 | -0.01 | 0.01 | -0.01 | 0.05 | -0.02 | 0.00 |
| Twice a month or more | 0.02 | 0.01 | 0.01 | 0.04 | 0.02 | 0.01 | 0.01 | 0.04 |
| Low support |  |  |  |  |  |  |  |  |
| No volunteering | 0.08 | 0.00 | 0.06 | 0.10 | 0.08 | 0.00 | 0.06 | 0.10 |
| Every few months or less often | -0.01 | 0.14 | -0.02 | 0.00 | -0.00 | 0.66 | -0.01 | 0.01 |
| Almost every month | -0.02 | 0.01 | -0.03 | -0.01 | -0.03 | 0.00 | -0.04 | -0.01 |
| Twice a month or more | -0.05 | 0.00 | -0.07 | -0.03 | -0.05 | 0.00 | -0.06 | -0.03 |
|  |  |  |  |  |  |  |  |  |
| **Baseline age** |  |  |  |  |  |  |  |  |
| No volunteering | 0.00 | 0.00 | 0.00 | 0.00 | 0.00 | 0.00 | 0.00 | 0.00 |
| Every few months or less often | -0.00 | 0.01 | -0.00 | -0.00 | -0.00 | 0.02 | -0.00 | -0.00 |
| Almost every month | -0.00 | 0.12 | -0.00 | 0.00 | -0.00 | 0.18 | -0.00 | 0.00 |
| Twice a month or more | -0.00 | 0.01 | -0.00 | -0.00 | -0.00 | 0.00 | -0.00 | -0.00 |
|  |  |  |  |  |  |  |  |  |
| **Sex** |  |  |  |  |  |  |  |  |
| Male (reference) | - | - | - | - | - | - | - | - |
| Female |  |  |  |  |  |  |  |  |
| No volunteering | -0.01 | 0.04 | -0.02 | -0.00 | -0.02 | 0.00 | -0.03 | -0.01 |
| Every few months or less often | -0.00 | 0.40 | -0.01 | 0.00 | 0.00 | 0.82 | -0.01 | 0.01 |
| Almost every month | 0.00 | 0.26 | -0.00 | 0.01 | 0.01 | 0.07 | -0.00 | 0.01 |
| Twice a month or more | 0.01 | 0.02 | 0.00 | 0.02 | 0.02 | 0.00 | 0.01 | 0.03 |
|  |  |  |  |  |  |  |  |  |
| **Living arrangement** |  |  |  |  |  |  |  |  |
| Live with partner (Yes) (reference) | - | - | - | - | - | - | - | - |
| Live with partner (No) |  |  |  |  |  |  |  |  |
| No volunteering | -0.00 | 0.49 | -0.02 | 0.01 | 0.01 | 0.25 | -0.01 | 0.02 |
| Every few months or less often | -0.00 | 0.53 | -0.01 | 0.01 | 0.00 | 0.65 | -0.01 | 0.01 |
| Almost every month | -0.00 | 0.60 | -0.01 | 0.01 | -0.01 | 0.01 | -0.02 | -0.00 |
| Twice a month or more | 0.01 | 0.10 | -0.00 | 0.02 | 0.00 | 1.00 | -0.01 | 0.01 |
|  |  |  |  |  |  |  |  |  |
| **Education** |  |  |  |  |  |  |  |  |
| Less than upper secondary education (reference) | - | - | - | - | - | - | - | - |
| Upper secondary and vocational training |  |  |  |  |  |  |  |  |
| No volunteering | -0.05 | 0.00 | -0.06 | -0.04 | -0.05 | 0.00 | -0.07 | -0.04 |
| Every few months or less often | 0.01 | 0.00 | 0.00 | 0.02 | 0.01 | 0.00 | 0.01 | 0.02 |
| Almost every month | 0.01 | 0.00 | 0.01 | 0.02 | 0.02 | 0.00 | 0.01 | 0.03 |
| Twice a month or more | 0.03 | 0.00 | 0.02 | 0.04 | 0.02 | 0.00 | 0.01 | 0.03 |
| Tertiary education |  |  |  |  |  |  |  |  |
| No volunteering | -0.10 | 0.00 | -0.11 | -0.08 | -0.10 | 0.00 | -0.11 | -0.08 |
| Every few months or less often | 0.02 | 0.00 | 0.01 | 0.03 | 0.02 | 0.00 | 0.01 | 0.03 |
| Almost every month | 0.02 | 0.00 | 0.01 | 0.03 | 0.02 | 0.00 | 0.01 | 0.03 |
| Twice a month or more | 0.05 | 0.00 | 0.04 | 0.07 | 0.06 | 0.00 | 0.04 | 0.07 |
|  |  |  |  |  |  |  |  |  |
| **Employment** |  |  |  |  |  |  |  |  |
| Retired (reference) | - | - | - | - | - | - | - | - |
| Full-time employed |  |  |  |  |  |  |  |  |
| No volunteering | 0.01 | 0.36 | -0.01 | 0.02 | -0.02 | 0.00 | -0.04 | -0.01 |
| Every few months or less often | 0.00 | 0.33 | -0.00 | 0.01 | 0.01 | 0.26 | -0.00 | 0.01 |
| Almost every month | -0.01 | 0.07 | -0.02 | 0.00 | 0.00 | 0.54 | -0.01 | 0.01 |
| Twice a month or more | -0.00 | 0.60 | -0.02 | 0.01 | 0.02 | 0.02 | 0.00 | 0.03 |
| Part-time employed |  |  |  |  |  |  |  |  |
| No volunteering | -0.01 | 0.70 | -0.05 | 0.03 | -0.03 | 0.24 | -0.07 | 0.02 |
| Every few months or less often | 0.01 | 0.39 | -0.01 | 0.04 | 0.01 | 0.55 | -0.02 | 0.03 |
| Almost every month | 0.00 | 0.80 | -0.02 | 0.03 | 0.01 | 0.31 | -0.01 | 0.04 |
| Twice a month or more | -0.01 | 0.70 | -0.04 | 0.03 | 0.01 | 0.75 | -0.03 | 0.04 |
| Unemployed |  |  |  |  |  |  |  |  |
| No volunteering | 0.01 | 0.66 | -0.03 | 0.04 | -0.00 | 1.00 | -0.03 | 0.03 |
| Every few months or less often | -0.01 | 0.53 | -0.02 | 0.01 | 0.01 | 0.61 | -0.02 | 0.03 |
| Almost every month | 0.01 | 0.25 | -0.01 | 0.04 | -0.00 | 0.99 | -0.02 | 0.02 |
| Twice a month or more | -0.02 | 0.24 | -0.04 | 0.01 | -0.01 | 0.72 | -0.03 | 0.02 |
| Permanently sick or disabled |  |  |  |  |  |  |  |  |
| No volunteering | 0.05 | 0.01 | 0.01 | 0.08 | 0.03 | 0.09 | -0.00 | 0.06 |
| Every few months or less often | -0.01 | 0.45 | -0.02 | 0.01 | 0.00 | 0.90 | -0.02 | 0.02 |
| Almost every month | -0.01 | 0.36 | -0.03 | 0.01 | -0.01 | 0.24 | -0.03 | 0.01 |
| Twice a month or more | -0.03 | 0.02 | -0.05 | -0.00 | -0.02 | 0.14 | -0.04 | 0.01 |
| Looking after home or family |  |  |  |  |  |  |  |  |
| No volunteering | 0.02 | 0.03 | 0.00 | 0.05 | 0.01 | 0.41 | -0.01 | 0.03 |
| Every few months or less often | -0.01 | 0.27 | -0.02 | 0.01 | 0.00 | 0.78 | -0.01 | 0.01 |
| Almost every month | 0.00 | 0.92 | -0.01 | 0.01 | 0.01 | 0.21 | -0.00 | 0.02 |
| Twice a month or more | -0.02 | 0.04 | -0.04 | -0.00 | -0.02 | 0.02 | -0.04 | -0.00 |
|  |  |  |  |  |  |  |  |  |
| **Household wealth** |  |  |  |  |  |  |  |  |
| Wealth (Q1 [lowest]) (reference) | - | - | - | - | - | - | - | - |
| Wealth Q2 |  |  |  |  |  |  |  |  |
| No volunteering | -0.00 | 0.89 | -0.02 | 0.02 | -0.01 | 0.59 | -0.02 | 0.01 |
| Every few months or less often | -0.01 | 0.14 | -0.02 | 0.00 | -0.00 | 0.52 | -0.01 | 0.01 |
| Almost every month | -0.00 | 0.83 | -0.01 | 0.01 | 0.00 | 0.48 | -0.01 | 0.01 |
| Twice a month or more | 0.01 | 0.21 | -0.01 | 0.02 | 0.00 | 0.56 | -0.01 | 0.02 |
| Wealth Q3 |  |  |  |  |  |  |  |  |
| No volunteering | -0.02 | 0.03 | -0.04 | -0.00 | -0.01 | 0.08 | -0.03 | 0.00 |
| Every few months or less often | 0.01 | 0.12 | -0.00 | 0.02 | 0.00 | 0.82 | -0.01 | 0.01 |
| Almost every month | -0.00 | 0.96 | -0.01 | 0.01 | 0.01 | 0.14 | -0.00 | 0.02 |
| Twice a month or more | 0.01 | 0.11 | -0.00 | 0.03 | 0.01 | 0.34 | -0.01 | 0.02 |
| Wealth Q4 |  |  |  |  |  |  |  |  |
| No volunteering | -0.02 | 0.01 | -0.04 | -0.01 | -0.03 | 0.00 | -0.05 | -0.01 |
| Every few months or less often | 0.01 | 0.24 | -0.00 | 0.01 | 0.01 | 0.10 | -0.00 | 0.02 |
| Almost every month | -0.00 | 0.96 | -0.01 | 0.01 | 0.01 | 0.11 | -0.00 | 0.02 |
| Twice a month or more | 0.02 | 0.01 | 0.00 | 0.03 | 0.02 | 0.02 | 0.00 | 0.03 |
| Wealth (Q5 [highest]) |  |  |  |  |  |  |  |  |
| No volunteering | -0.03 | 0.00 | -0.05 | -0.01 | -0.05 | 0.00 | -0.06 | -0.03 |
| Every few months or less often | 0.01 | 0.23 | -0.00 | 0.01 | 0.01 | 0.14 | -0.00 | 0.02 |
| Almost every month | 0.01 | 0.17 | -0.00 | 0.02 | 0.00 | 0.26 | -0.00 | 0.01 |
| Twice a month or more | 0.02 | 0.01 | 0.00 | 0.03 | 0.03 | 0.00 | 0.02 | 0.05 |
|  |  |  |  |  |  |  |  |  |
| **Limiting illness** |  |  |  |  |  |  |  |  |
| No illness (reference) | - | - | - | - | - | - | - | - |
| Not limiting illness |  |  |  |  |  |  |  |  |
| No volunteering | -0.01 | 0.22 | -0.02 | 0.01 | -0.00 | 0.83 | -0.02 | 0.01 |
| Every few months or less often | 0.00 | 0.69 | -0.01 | 0.01 | -0.00 | 0.28 | -0.01 | 0.00 |
| Almost every month | -0.00 | 0.87 | -0.01 | 0.01 | 0.01 | 0.17 | -0.00 | 0.01 |
| Twice a month or more | 0.01 | 0.17 | -0.00 | 0.02 | 0.00 | 0.96 | -0.01 | 0.01 |
| Limiting illness |  |  |  |  |  |  |  |  |
| No volunteering | 0.01 | 0.03 | 0.00 | 0.03 | 0.02 | 0.01 | 0.01 | 0.03 |
| Every few months or less often | -0.00 | 0.54 | -0.01 | 0.01 | -0.01 | 0.03 | -0.02 | -0.00 |
| Almost every month | -0.00 | 0.54 | -0.01 | 0.01 | -0.00 | 0.29 | -0.01 | 0.00 |
| Twice a month or more | -0.01 | 0.07 | -0.02 | 0.00 | -0.01 | 0.22 | -0.02 | 0.00 |
|  |  |  |  |  |  |  |  |  |
| **Mental health symptoms** |  |  |  |  |  |  |  |  |
| Mental health caseness (No) (reference) | - | - | - | - | - | - | - | - |
| Mental health caseness (Yes) |  |  |  |  |  |  |  |  |
| No volunteering | 0.01 | 0.09 | -0.00 | 0.02 | 0.01 | 0.35 | -0.01 | 0.02 |
| Every few months or less often | -0.00 | 0.89 | -0.01 | 0.01 | -0.00 | 0.69 | -0.01 | 0.01 |
| Almost every month | -0.01 | 0.00 | -0.02 | -0.01 | -0.00 | 0.22 | -0.01 | 0.00 |
| Twice a month or more | 0.00 | 0.61 | -0.01 | 0.01 | -0.00 | 0.95 | -0.01 | 0.01 |

**Supplementary Table 2.** Care status and volunteering frequency (relative risk ratios), 2014 and 2019 results (N= 15,555)

|  | **2015** | | | | | | | | **2019** | | | | | | | |
| --- | --- | --- | --- | --- | --- | --- | --- | --- | --- | --- | --- | --- | --- | --- | --- | --- |
|  | Unadjusted | | | | Adjusted | | | | Unadjusted | | | | Adjusted | | | |
| Volunteering frequency | RRR | p-value | 95% CI | | RRR | p-value | 95% CI | | RRR | p-value | 95% CI | | RRR | p-value | 95% CI | |
| **No volunteering** (base outcome) | - | - | - | - | - | - | - | - | - | - | - | - | - | - | - | - |
| **Every few months or less often** |  |  |  |  |  |  |  |  |  |  |  |  |  |  |  |  |
| Non-carer (Reference) |  |  |  |  |  |  |  |  |  |  |  |  |  |  |  |  |
| Carer | 1.39 | 0.02 | 1.05 | 1.85 | 1.15 | 0.36 | 0.86 | 1.53 | 1.68 | 0.00 | 1.30 | 2.17 | 1.35 | 0.03 | 1.03 | 1.75 |
| Former carer | 1.43 | 0.00 | 1.15 | 1.78 | 1.28 | 0.03 | 1.03 | 1.61 | 1.03 | 0.82 | 0.81 | 1.31 | 0.91 | 0.44 | 0.71 | 1.16 |
| **Almost every month** |  |  |  |  |  |  |  |  |  |  |  |  |  |  |  |  |
| Non-carer (Reference) |  |  |  |  |  |  |  |  |  |  |  |  |  |  |  |  |
| Carer | 1.99 | 0.00 | 1.57 | 2.53 | 1.79 | 0.00 | 1.39 | 2.31 | 1.44 | 0.01 | 1.09 | 1.90 | 1.13 | 0.41 | 0.85 | 1.51 |
| Former carer | 1.32 | 0.01 | 1.07 | 1.65 | 1.21 | 0.10 | 0.96 | 1.52 | 1.02 | 0.89 | 0.80 | 1.30 | 0.89 | 0.37 | 0.69 | 1.15 |
| **Twice a month or more** |  |  |  |  |  |  |  |  |  |  |  |  |  |  |  |  |
| Non-carer (Reference) |  |  |  |  |  |  |  |  |  |  |  |  |  |  |  |  |
| Carer | 2.67 | 0.00 | 2.30 | 3.10 | 2.17 | 0.00 | 1.81 | 2.60 | 2.62 | 0.00 | 2.25 | 3.04 | 1.90 | 0.00 | 1.60 | 2.26 |
| Former carer | 1.58 | 0.00 | 1.38 | 1.82 | 1.27 | 0.00 | 1.08 | 1.50 | 1.69 | 0.00 | 1.47 | 1.94 | 1.37 | 0.00 | 1.17 | 1.60 |

Adjusted model controls for outcome at baseline, care regimes, age, gender, living with partner, education, employment status, household wealth, longstanding limiting illness, and depression symptomatology.

**Supplementary Table 3.** Care frequency and volunteering frequency (relative risk ratios), 2015 and 2019 results (N=15,555)

|  | **2015** | | | | | | | | **2019** | | | | | | | |
| --- | --- | --- | --- | --- | --- | --- | --- | --- | --- | --- | --- | --- | --- | --- | --- | --- |
|  | Unadjusted | | | | Adjusted | | | | Unadjusted | | | | Adjusted | | | |
| Volunteering frequency | RRR | p-value | 95% CI | | RRR | p-value | 95% CI | | RRR | p-value | 95% CI | | RRR | p-value | 95% CI | |
| **No volunteering (**base outcome) | - | - | - | - | - | - | - | - | - | - | - | - | - | - | - | - |
| **Every few months or less often** |  |  |  |  |  |  |  |  |  |  |  |  |  |  |  |  |
| Non-carer (Reference) | - | - | - | - | - | - | - | - | - | - | - | - | - | - | - | - |
| Former carer | 1.43 | 0.00 | 1.15 | 1.78 | 1.28 | 0.03 | 1.03 | 1.61 | 1.03 | 0.82 | 0.81 | 1.31 | 0.91 | 0.44 | 0.71 | 1.16 |
| Daily in household | 1.32 | 0.21 | 0.86 | 2.03 | 1.23 | 0.35 | 0.79 | 1.92 | 1.51 | 0.04 | 1.02 | 2.23 | 1.41 | 0.10 | 0.94 | 2.11 |
| Daily outside household | 1.27 | 0.42 | 0.71 | 2.29 | 0.96 | 0.89 | 0.52 | 1.75 | 1.68 | 0.05 | 1.00 | 2.81 | 1.23 | 0.45 | 0.72 | 2.09 |
| Weekly outside household | 1.54 | 0.07 | 0.96 | 2.47 | 1.14 | 0.60 | 0.70 | 1.85 | 2.09 | 0.00 | 1.40 | 3.14 | 1.49 | 0.06 | 0.98 | 2.27 |
| Monthly | 1.64 | 0.34 | 0.59 | 4.55 | 1.41 | 0.52 | 0.50 | 3.96 | 1.01 | 0.98 | 0.32 | 3.23 | 0.77 | 0.66 | 0.24 | 2.48 |
| **Almost every month** |  |  |  |  |  |  |  |  |  |  |  |  |  |  |  |  |
| Non-carer (Reference) | - | - | - | - | - | - | - | - | - | - | - | - | - | - | - | - |
| Former carer | 1.32 | 0.01 | 1.07 | 1.65 | 1.21 | 0.10 | 0.96 | 1.52 | 1.02 | 0.89 | 0.80 | 1.30 | 0.89 | 0.37 | 0.69 | 1.15 |
| Daily in household | 1.87 | 0.00 | 1.31 | 2.68 | 1.81 | 0.00 | 1.24 | 2.64 | 1.06 | 0.80 | 0.66 | 1.70 | 0.93 | 0.76 | 0.57 | 1.51 |
| Daily outside household | 1.09 | 0.79 | 0.59 | 2.01 | 0.91 | 0.76 | 0.48 | 1.71 | 1.53 | 0.13 | 0.88 | 2.65 | 1.11 | 0.71 | 0.63 | 1.97 |
| Weekly outside household | 2.27 | 0.00 | 1.54 | 3.34 | 1.91 | 0.00 | 1.27 | 2.87 | 1.45 | 0.13 | 0.89 | 2.36 | 1.05 | 0.83 | 0.64 | 1.74 |
| Monthly | 4.98 | 0.00 | 2.71 | 9.16 | 4.66 | 0.00 | 2.41 | 9.00 | 3.51 | 0.00 | 1.80 | 6.88 | 2.45 | 0.01 | 1.19 | 5.03 |
| **Twice a month or more** |  |  |  |  |  |  |  |  |  |  |  |  |  |  |  |  |
| Non-carer (Reference) | - | - | - | - | - | - | - | - | - | - | - | - | - | - | - | - |
| Former carer | 1.58 | 0.00 | 1.38 | 1.82 | 1.27 | 0.00 | 1.08 | 1.50 | 1.69 | 0.00 | 1.47 | 1.94 | 1.37 | 0.00 | 1.17 | 1.60 |
| Daily in household | 1.91 | 0.00 | 1.49 | 2.44 | 1.74 | 0.00 | 1.30 | 2.33 | 1.89 | 0.00 | 1.48 | 2.42 | 1.64 | 0.00 | 1.24 | 2.18 |
| Daily outside household | 2.66 | 0.00 | 1.99 | 3.55 | 2.03 | 0.00 | 1.42 | 2.89 | 2.73 | 0.00 | 2.04 | 3.66 | 1.84 | 0.00 | 1.31 | 2.59 |
| Weekly outside household | 3.80 | 0.00 | 3.03 | 4.76 | 2.71 | 0.00 | 2.07 | 3.56 | 3.91 | 0.00 | 3.13 | 4.89 | 2.45 | 0.00 | 1.89 | 3.17 |
| Monthly | 2.37 | 0.00 | 1.32 | 4.26 | 2.36 | 0.01 | 1.21 | 4.61 | 1.17 | 0.67 | 0.56 | 2.45 | 0.82 | 0.63 | 0.37 | 1.82 |

Adjusted model controls for outcome at baseline, care regimes, age, gender, living with partner, education, employment status, household wealth, longstanding limiting illness, and depression symptomatology.

**Supplementary Table 4.** Care recipient and volunteering frequency (relative risk ratios), 2015 and 2019 results (N=15,555)

|  | | | | | | | | | | | | | | | | |
| --- | --- | --- | --- | --- | --- | --- | --- | --- | --- | --- | --- | --- | --- | --- | --- | --- |
|  | **2015** | | | | | | | | **2019** | | | | | | | |
|  | Unadjusted | | | | Adjusted | | | | Unadjusted | | | | Adjusted | | | |
| Volunteering frequency | RRR | p-value | 95% CI | | RRR | p-value | 95% CI | | RRR | p-value | 95% CI | | RRR | p-value | 95% CI | |
| **No volunteering** (base outcome) | - | - | - | - | - | - | - | - | - | - | - | - | - | - | - | - |
| **Every few months or less often** |  |  |  |  |  |  |  |  |  |  |  |  |  |  |  |  |
| Non-carer (Reference) | - | - | - | - | - | - | - | - | - | - | - | - | - | - | - | - |
| Former carer | 1.43 | 0.00 | 1.15 | 1.78 | 1.28 | 0.03 | 1.03 | 1.61 | 1.03 | 0.82 | 0.81 | 1.31 | 0.91 | 0.44 | 0.71 | 1.16 |
| Partner | 1.14 | 0.61 | 0.68 | 1.90 | 1.08 | 0.77 | 0.64 | 1.82 | 1.24 | 0.38 | 0.77 | 2.01 | 1.23 | 0.41 | 0.75 | 2.02 |
| Child | 0.86 | 0.69 | 0.40 | 1.83 | 0.81 | 0.60 | 0.37 | 1.76 | 2.54 | 0.00 | 1.60 | 4.02 | 2.51 | 0.00 | 1.55 | 4.06 |
| Parent | 1.82 | 0.01 | 1.16 | 2.87 | 1.37 | 0.19 | 0.86 | 2.19 | 2.23 | 0.00 | 1.49 | 3.35 | 1.54 | 0.05 | 1.01 | 2.35 |
| Parent-in-law | 1.16 | 0.77 | 0.42 | 3.19 | 0.83 | 0.72 | 0.30 | 2.31 | 1.89 | 0.11 | 0.87 | 4.11 | 1.29 | 0.53 | 0.58 | 2.86 |
| Other relative | 1.29 | 0.55 | 0.56 | 2.95 | 1.06 | 0.89 | 0.46 | 2.46 | 0.79 | 0.65 | 0.29 | 2.16 | 0.59 | 0.31 | 0.21 | 1.64 |
| Non-relative | 2.50 | 0.00 | 1.50 | 4.16 | 2.29 | 0.00 | 1.36 | 3.86 | 2.37 | 0.00 | 1.48 | 3.79 | 1.93 | 0.01 | 1.19 | 3.14 |
| **Almost every month** |  |  |  |  |  |  |  |  |  |  |  |  |  |  |  |  |
| Non-carer (Reference) | - | - | - | - | - | - | - | - | - | - | - | - | - | - | - | - |
| Former carer | 1.32 | 0.01 | 1.07 | 1.65 | 1.21 | 0.10 | 0.96 | 1.52 | 1.02 | 0.89 | 0.80 | 1.30 | 0.89 | 0.37 | 0.69 | 1.15 |
| Partner | 1.60 | 0.03 | 1.04 | 2.44 | 1.52 | 0.07 | 0.97 | 2.39 | 1.15 | 0.60 | 0.69 | 1.91 | 1.05 | 0.86 | 0.62 | 1.78 |
| Child | 1.82 | 0.02 | 1.09 | 3.06 | 1.87 | 0.03 | 1.08 | 3.24 | 0.88 | 0.74 | 0.41 | 1.88 | 0.86 | 0.70 | 0.39 | 1.87 |
| Parent | 2.18 | 0.00 | 1.45 | 3.28 | 1.89 | 0.00 | 1.22 | 2.91 | 1.72 | 0.02 | 1.08 | 2.74 | 1.19 | 0.48 | 0.73 | 1.94 |
| Parent-in-law | 2.17 | 0.04 | 1.04 | 4.51 | 1.92 | 0.10 | 0.89 | 4.14 | 2.25 | 0.03 | 1.08 | 4.67 | 1.56 | 0.26 | 0.72 | 3.34 |
| Other relative | 1.60 | 0.20 | 0.78 | 3.30 | 1.34 | 0.45 | 0.63 | 2.89 | 1.24 | 0.61 | 0.54 | 2.82 | 0.94 | 0.89 | 0.40 | 2.21 |
| Non-relative | 4.39 | 0.00 | 2.97 | 6.49 | 4.23 | 0.00 | 2.79 | 6.42 | 1.85 | 0.02 | 1.08 | 3.15 | 1.43 | 0.20 | 0.82 | 2.49 |
| **Twice a month or more** |  |  |  |  |  |  |  |  |  |  |  |  |  |  |  |  |
| Non-carer (Reference) | - | - | - | - | - | - | - | - | - | - | - | - | - | - | - | - |
| Former carer | 1.58 | 0.00 | 1.38 | 1.82 | 1.27 | 0.00 | 1.08 | 1.50 | 1.69 | 0.00 | 1.47 | 1.94 | 1.37 | 0.00 | 1.17 | 1.60 |
| Partner | 1.94 | 0.00 | 1.48 | 2.54 | 1.75 | 0.00 | 1.26 | 2.43 | 2.19 | 0.00 | 1.68 | 2.84 | 2.09 | 0.00 | 1.54 | 2.83 |
| Child | 1.46 | 0.06 | 0.98 | 2.17 | 1.49 | 0.09 | 0.94 | 2.35 | 1.52 | 0.04 | 1.03 | 2.26 | 1.46 | 0.09 | 0.94 | 2.28 |
| Parent | 2.03 | 0.00 | 1.52 | 2.72 | 1.59 | 0.01 | 1.13 | 2.25 | 1.97 | 0.00 | 1.47 | 2.66 | 1.17 | 0.35 | 0.84 | 1.64 |
| Parent-in-law | 2.75 | 0.00 | 1.72 | 4.39 | 2.40 | 0.00 | 1.38 | 4.17 | 2.00 | 0.01 | 1.18 | 3.37 | 1.25 | 0.46 | 0.69 | 2.25 |
| Other relative | 1.77 | 0.02 | 1.09 | 2.86 | 1.31 | 0.35 | 0.74 | 2.33 | 1.63 | 0.05 | 1.00 | 2.67 | 1.07 | 0.82 | 0.61 | 1.87 |
| Non-relative | 6.11 | 0.00 | 4.73 | 7.90 | 5.38 | 0.00 | 3.92 | 7.37 | 4.27 | 0.00 | 3.28 | 5.57 | 3.04 | 0.00 | 2.22 | 4.16 |

Adjusted model controls for outcome at baseline, care regimes, age, gender, living with partner, education, employment status, household wealth, longstanding limiting illness, and depression symptomatology.

**Supplementary Table 5.** Care status and group membership adjusted models, 2015 and 2019 results (N=14,809)

|  | **2015** | | | | **2019** | | | |
| --- | --- | --- | --- | --- | --- | --- | --- | --- |
|  | OR | p-value | 95% CI | | OR | p-value | 95% CI | |
| **Carer status** |  |  |  |  |  |  |  |  |
| Non-carer (reference) | - | - | - | - | - | - | - | - |
| Carer | 1.39 | 0.00 | 1.21 | 1.59 | 1.21 | 0.01 | 1.06 | 1.39 |
| Former carer | 1.16 | 0.01 | 1.04 | 1.30 | 1.02 | 0.77 | 0.91 | 1.14 |
| **Baseline group membership** |  |  |  |  |  |  |  |  |
| No group membership (reference) | - | - | - | - | - | - | - | - |
| Member of 1-3 groups | 6.10 | 0.00 | 5.62 | 6.61 | 4.46 | 0.00 | 4.12 | 4.84 |
| **Regime** |  |  |  |  |  |  |  |  |
| High support (reference) | - | - | - | - | - | - | - | - |
| Medum support | 0.43 | 0.00 | 0.38 | 0.50 | 0.47 | 0.00 | 0.41 | 0.53 |
| Low support | 0.26 | 0.00 | 0.22 | 0.30 | 0.25 | 0.00 | 0.21 | 0.28 |
| **Baseline age** | 0.98 | 0.00 | 0.97 | 0.99 | 0.96 | 0.00 | 0.95 | 0.97 |
| **Sex** |  |  |  |  |  |  |  |  |
| Male (reference) | - | - | - | - | - | - | - | - |
| Female | 1.15 | 0.00 | 1.06 | 1.25 | 1.20 | 0.00 | 1.10 | 1.30 |
| **Living arrangement** |  |  |  |  |  |  |  |  |
| Live with partner (Yes) (reference) | - | - | - | - | - | - | - | - |
| Live with partner (No) | 0.93 | 0.18 | 0.84 | 1.03 | 0.95 | 0.30 | 0.86 | 1.05 |
| **Education** |  |  |  |  |  |  |  |  |
| Less than upper secondary education (reference) | - | - | - | - | - | - | - | - |
| Upper secondary and vocational training | 1.55 | 0.00 | 1.41 | 1.71 | 1.51 | 0.00 | 1.37 | 1.66 |
| Tertiary education | 2.37 | 0.00 | 2.11 | 2.65 | 2.27 | 0.00 | 2.03 | 2.54 |
| **Employment** |  |  |  |  |  |  |  |  |
| Retired (reference) | - | - | - | - | - | - | - | - |
| Full-time employed or self-employed | 0.91 | 0.14 | 0.81 | 1.03 | 0.85 | 0.01 | 0.76 | 0.96 |
| Part-time employed or self-employed | 0.93 | 0.67 | 0.67 | 1.30 | 0.94 | 0.70 | 0.68 | 1.30 |
| Unemployed | 0.64 | 0.00 | 0.49 | 0.83 | 0.68 | 0.00 | 0.53 | 0.87 |
| Permanently sick or disabled | 0.47 | 0.00 | 0.35 | 0.62 | 0.50 | 0.00 | 0.39 | 0.66 |
| Looking after home or family | 0.71 | 0.00 | 0.60 | 0.84 | 0.59 | 0.00 | 0.50 | 0.71 |
| **Household wealth** |  |  |  |  |  |  |  |  |
| Wealth Q1 (lowest) (reference) | - | - | - | - | - | - | - | - |
| Q2 | 1.04 | 0.55 | 0.91 | 1.20 | 1.18 | 0.02 | 1.03 | 1.36 |
| Q3 | 1.11 | 0.09 | 0.98 | 1.26 | 1.21 | 0.00 | 1.07 | 1.38 |
| Q4 | 1.27 | 0.00 | 1.12 | 1.43 | 1.52 | 0.00 | 1.34 | 1.72 |
| Q5 (highest) | 1.33 | 0.00 | 1.18 | 1.51 | 1.46 | 0.00 | 1.29 | 1.65 |
| **Limiting illness** |  |  |  |  |  |  |  |  |
| No illness (reference) | - | - | - | - | - | - | - | - |
| Not limiting illness | 1.03 | 0.61 | 0.92 | 1.15 | 0.98 | 0.75 | 0.88 | 1.09 |
| Limiting illness | 0.90 | 0.03 | 0.82 | 0.99 | 0.85 | 0.00 | 0.77 | 0.93 |
| **Mental health sypmtoms** |  |  |  |  |  |  |  |  |
| Mental health caseness (No) (reference) | - | - | - | - | - | - | - | - |
| Mental health caseness (Yes) | 0.85 | 0.00 | 0.77 | 0.93 | 0.85 | 0.00 | 0.78 | 0.94 |

**Supplementary Table 6.** Care status by individual group membership, 2015 and 2019 results (N=14,809)

|  | | **2015** | | | **2019** | | |
| --- | --- | --- | --- | --- | --- | --- | --- |
|  | | Not mentioned | Mentioned | | Not mentioned | Mentioned | |
| **Education groups** |  | N | N | % | N | N | % |
|  | Non-carer | 10,168 | 1,171 | 10.33 | 10,323 | 1,016 | 8.96 |
|  | Carer | 1,072 | 227 | 17.47 | 1,093 | 206 | 15.86 |
|  | Former carer | 1,887 | 284 | 13.08 | 1,935 | 236 | 10.87 |
|  | Total | 13,127 | 1,682 | 11.36 | 13,351 | 1,458 | 9.85 |
| **Exercise groups** |  |  |  |  |  |  |  |
|  | Non-carer | 8,229 | 3,110 | 27.43 | 8,268 | 3,071 | 27.08 |
|  | Carer | 846 | 453 | 34.87 | 861 | 438 | 33.72 |
|  | Former carer | 1,536 | 635 | 29.25 | 1,559 | 612 | 28.19 |
|  | Total | 10,611 | 4,198 | 28.35 | 10,688 | 4,121 | 27.83 |
| **Political groups** |  |  |  |  |  |  |  |
|  | Non-carer | 10,519 | 820 | 7.23 | 10,644 | 695 | 6.13 |
|  | Carer | 1,162 | 137 | 10.55 | 1,198 | 101 | 7.78 |
|  | Former carer | 1,991 | 180 | 8.29 | 2,021 | 150 | 6.91 |
|  | Total | 13,672 | 1,137 | 7.68 | 13,863 | 946 | 6.39 |

Education groups: Chi2 for 2015 and 2019 p<0.001; Exercise groups Chi2 for 2015 and 2019 p<0.001; Political groups Chi2 for 2015 and 2019 p<0.05

**Supplementary Table 7.** Care status and volunteering frequency (participants with outcome data in 2015) (N =30,828)

|  | **Unadjusted** | | | | **Adjusted** | | | |
| --- | --- | --- | --- | --- | --- | --- | --- | --- |
|  | AME | p-value | 95% CI | | AME | p-value | 95% CI | |
| **Non-care** |  |  |  |  |  |  |  |  |
| Reference | - | - | - | - | - | - | - | - |
| **Carer** |  |  |  |  |  |  |  |  |
| No volunteering | -0.14 | 0.00 | -0.16 | -0.12 | -0.07 | 0.00 | -0.09 | -0.06 |
| Every few months or less often | 0.01 | 0.01 | 0.00 | 0.02 | 0.00 | 0.29 | -0.00 | 0.01 |
| Almost every month | 0.03 | 0.00 | 0.02 | 0.04 | 0.02 | 0.00 | 0.01 | 0.03 |
| Twice a month or more | 0.10 | 0.00 | 0.08 | 0.11 | 0.05 | 0.00 | 0.04 | 0.06 |
| **Former carer** |  |  |  |  |  |  |  |  |
| No volunteering | -0.06 | 0.00 | -0.07 | -0.04 | -0.03 | 0.00 | -0.04 | -0.02 |
| Every few months or less often | 0.01 | 0.00 | 0.00 | 0.02 | 0.01 | 0.05 | -0.00 | 0.01 |
| Almost every month | 0.01 | 0.01 | 0.00 | 0.02 | 0.00 | 0.11 | -0.00 | 0.01 |
| Twice a month or more | 0.04 | 0.00 | 0.03 | 0.05 | 0.01 | 0.00 | 0.01 | 0.02 |

Adjusted model controls for outcome at baseline, care regimes, age, gender, living with partner, education, employment status, household wealth, longstanding limiting illness, and depression symptomatology.

**Supplementary Table 8.** Care frequency and volunteering frequency (participants with outcome data in 2015) (N =30,828)

|  | **Unadjusted** | | | | **Adjusted** | | | |
| --- | --- | --- | --- | --- | --- | --- | --- | --- |
|  | AME | p-value | 905% CI | | AME | p-value | 905% CI | |
| **Non-care** |  |  |  |  |  |  |  |  |
| Reference | - | - | - | - | - | - | - | - |
| **Former carer** |  |  |  |  |  |  |  |  |
| No volunteering | -0.06 | 0.00 | -0.07 | -0.04 | -0.03 | 0.00 | -0.04 | -0.02 |
| Every few months or less often | 0.01 | 0.00 | 0.00 | 0.02 | 0.01 | 0.05 | -0.00 | 0.01 |
| Almost every month | 0.01 | 0.01 | 0.00 | 0.02 | 0.00 | 0.11 | -0.00 | 0.01 |
| Twice a month or more | 0.04 | 0.00 | 0.03 | 0.05 | 0.01 | 0.00 | 0.01 | 0.02 |
| **Daily care in household** |  |  |  |  |  |  |  |  |
| No volunteering | -0.10 | 0.00 | -0.12 | -0.07 | -0.06 | 0.00 | -0.08 | -0.03 |
| Every few months or less often | 0.00 | 0.42 | -0.01 | 0.02 | 0.00 | 0.79 | -0.01 | 0.01 |
| Almost every month | 0.03 | 0.00 | 0.01 | 0.04 | 0.02 | 0.00 | 0.01 | 0.03 |
| Twice a month or more | 0.06 | 0.00 | 0.04 | 0.09 | 0.04 | 0.00 | 0.02 | 0.05 |
| **Daily care outside household** |  |  |  |  |  |  |  |  |
| No volunteering | -0.11 | 0.00 | -0.14 | -0.07 | -0.05 | 0.00 | -0.08 | -0.03 |
| Every few months or less often | -0.00 | 0.96 | -0.01 | 0.01 | -0.01 | 0.33 | -0.02 | 0.01 |
| Almost every month | 0.01 | 0.29 | -0.01 | 0.02 | 0.00 | 0.85 | -0.01 | 0.01 |
| Twice a month or more | 0.10 | 0.00 | 0.07 | 0.13 | 0.06 | 0.00 | 0.04 | 0.08 |
| **Weekly care outside household** |  |  |  |  |  |  |  |  |
| No volunteering | -0.20 | 0.00 | -0.24 | -0.17 | -0.09 | 0.00 | -0.12 | -0.07 |
| Every few months or less often | 0.01 | 0.08 | -0.00 | 0.03 | 0.00 | 0.57 | -0.01 | 0.02 |
| Almost every month | 0.04 | 0.00 | 0.02 | 0.05 | 0.02 | 0.01 | 0.01 | 0.04 |
| Twice a month or more | 0.15 | 0.00 | 0.12 | 0.18 | 0.07 | 0.00 | 0.05 | 0.09 |
| **Monthly care** |  |  |  |  |  |  |  |  |
| No volunteering | -0.21 | 0.00 | -0.28 | -0.14 | -0.13 | 0.00 | -0.19 | -0.08 |
| Every few months or less often | 0.07 | 0.00 | 0.02 | 0.11 | 0.05 | 0.01 | 0.01 | 0.09 |
| Almost every month | 0.08 | 0.00 | 0.03 | 0.12 | 0.05 | 0.00 | 0.02 | 0.09 |
| Twice a month or more | 0.07 | 0.01 | 0.02 | 0.12 | 0.03 | 0.08 | -0.00 | 0.07 |

Adjusted model controls for outcome at baseline, care regimes, age, gender, living with partner, education, employment status, household wealth, longstanding limiting illness, and depression symptomatology.

**Supplementary Table 9.** Relationship to care recipient and volunteering frequency (participants with outcome data in 2015) (N =30,828)

|  | **Unadjusted** | | | | **Adjusted** | | | |
| --- | --- | --- | --- | --- | --- | --- | --- | --- |
|  | AME | p-value | 905% CI | | AME | p-value | 905% CI | |
| **Non-care** |  |  |  |  |  |  |  |  |
| Reference | - | - | - | - | - | - | - | - |
| **Former carer** |  |  |  |  |  |  |  |  |
| No volunteering | -0.06 | 0.00 | -0.07 | -0.04 | -0.03 | 0.00 | -0.04 | -0.02 |
| Every few months or less often | 0.01 | 0.00 | 0.00 | 0.02 | 0.01 | 0.05 | -0.00 | 0.01 |
| Almost every month | 0.01 | 0.01 | 0.00 | 0.02 | 0.00 | 0.11 | -0.00 | 0.01 |
| Twice a month or more | 0.04 | 0.00 | 0.03 | 0.05 | 0.01 | 0.00 | 0.01 | 0.02 |
| **Partner** |  |  |  |  |  |  |  |  |
| No volunteering | -0.09 | 0.00 | -0.13 | -0.06 | -0.06 | 0.00 | -0.08 | -0.03 |
| Every few months or less often | 0.01 | 0.39 | -0.01 | 0.02 | 0.00 | 0.65 | -0.01 | 0.02 |
| Almost every month | 0.03 | 0.00 | 0.01 | 0.04 | 0.02 | 0.01 | 0.00 | 0.03 |
| Twice a month or more | 0.06 | 0.00 | 0.04 | 0.09 | 0.04 | 0.00 | 0.02 | 0.06 |
| **Child** |  |  |  |  |  |  |  |  |
| No volunteering | -0.06 | 0.00 | -0.10 | -0.03 | -0.05 | 0.00 | -0.08 | -0.02 |
| Every few months or less often | -0.01 | 0.38 | -0.02 | 0.01 | -0.01 | 0.24 | -0.02 | 0.01 |
| Almost every month | 0.03 | 0.01 | 0.01 | 0.05 | 0.03 | 0.01 | 0.01 | 0.05 |
| Twice a month or more | 0.04 | 0.01 | 0.01 | 0.07 | 0.03 | 0.02 | 0.00 | 0.05 |
| **Parent** |  |  |  |  |  |  |  |  |
| No volunteering | -0.12 | 0.00 | -0.15 | -0.09 | -0.04 | 0.00 | -0.07 | -0.02 |
| Every few months or less often | 0.02 | 0.02 | 0.00 | 0.04 | 0.01 | 0.35 | -0.01 | 0.02 |
| Almost every month | 0.03 | 0.00 | 0.01 | 0.05 | 0.02 | 0.04 | 0.00 | 0.03 |
| Twice a month or more | 0.07 | 0.00 | 0.04 | 0.09 | 0.02 | 0.01 | 0.01 | 0.04 |
| **Parent-in-law** |  |  |  |  |  |  |  |  |
| No volunteering | -0.14 | 0.00 | -0.20 | -0.08 | -0.08 | 0.00 | -0.12 | -0.03 |
| Every few months or less often | 0.01 | 0.45 | -0.02 | 0.04 | 0.00 | 0.99 | -0.02 | 0.02 |
| Almost every month | 0.04 | 0.03 | 0.00 | 0.07 | 0.02 | 0.09 | -0.00 | 0.05 |
| Twice a month or more | 0.09 | 0.00 | 0.04 | 0.14 | 0.05 | 0.01 | 0.01 | 0.09 |
| **Other relative** |  |  |  |  |  |  |  |  |
| No volunteering | -0.12 | 0.00 | -0.17 | -0.07 | -0.06 | 0.01 | -0.10 | -0.02 |
| Every few months or less often | 0.01 | 0.65 | -0.02 | 0.03 | -0.00 | 0.93 | -0.02 | 0.02 |
| Almost every month | 0.04 | 0.01 | 0.01 | 0.07 | 0.02 | 0.06 | -0.00 | 0.05 |
| Twice a month or more | 0.08 | 0.00 | 0.03 | 0.12 | 0.03 | 0.04 | 0.00 | 0.06 |
| **Non-relative** |  |  |  |  |  |  |  |  |
| No volunteering | -0.31 | 0.00 | -0.35 | -0.26 | -0.19 | 0.00 | -0.22 | -0.15 |
| Every few months or less often | 0.03 | 0.01 | 0.01 | 0.05 | 0.02 | 0.03 | 0.00 | 0.04 |
| Almost every month | 0.05 | 0.00 | 0.03 | 0.08 | 0.04 | 0.00 | 0.02 | 0.06 |
| Twice a month or more | 0.23 | 0.00 | 0.19 | 0.27 | 0.13 | 0.00 | 0.10 | 0.16 |

Adjusted model controls for outcome at baseline, care regimes, age, gender, living with partner, education, employment status, household wealth, longstanding limiting illness, and depression symptomatology.

**Supplementary Table 10.** Care status and group membership (participants with outcome data in 2015) (N=30,097)

|  | Unadjusted | | | | Adjusted | | | |
| --- | --- | --- | --- | --- | --- | --- | --- | --- |
| Group membership | OR | p-value | 95% CI | | OR | p-value | 95% CI | |
| Non-carer (Reference) | - | - | - | - | - | - | - | - |
| Carer | 1.65 | 0.00 | 1.51 | 1.81 | 1.31 | 0.00 | 1.18 | 1.46 |
| Former carer | 1.26 | 0.00 | 1.17 | 1.35 | 1.17 | 0.00 | 1.08 | 1.27 |

Adjusted model controls for outcome at baseline, care regimes, age, gender, living with partner, education, employment status, household wealth, longstanding limiting illness, and depression symptomatology.

**Supplementary Table 11.** Care frequency and group membership (participants with outcome data in 2015) (N=30,097)

|  | Unadjusted | | | | Adjusted | | | |
| --- | --- | --- | --- | --- | --- | --- | --- | --- |
| Group membership | OR | p-value | 95% CI | | OR | p-value | 95% CI | |
| Non-carer (Reference) | - | - | - | - | - | - | - | - |
| Former carer | 1.26 | 0.00 | 1.17 | 1.35 | 1.17 | 0.00 | 1.08 | 1.28 |
| Daily in household | 1.31 | 0.00 | 1.14 | 1.51 | 1.19 | 0.04 | 1.01 | 1.41 |
| Daily outside household | 1.50 | 0.00 | 1.26 | 1.78 | 1.26 | 0.03 | 1.03 | 1.54 |
| Weekly outside household | 2.10 | 0.00 | 1.79 | 2.45 | 1.42 | 0.00 | 1.18 | 1.70 |
| Monthly | 2.77 | 0.00 | 2.05 | 3.73 | 1.68 | 0.00 | 1.18 | 2.40 |

Adjusted model controls for outcome at baseline, care regimes, age, gender, living with partner, education, employment status, household wealth, longstanding limiting illness, and depression symptomatology.

**Supplementary Table 12.** Relationship to care recipient and group membership (participants with outcome data in 2015) (N=30,097)

|  | Unadjusted | | | | Adjusted | | | |
| --- | --- | --- | --- | --- | --- | --- | --- | --- |
| Group membership | OR | p-value | 95% CI | | OR | p-value | 95% CI | |
| Non-carer (Reference) | - | - | - | - | - | - | - | - |
| Former carer | 1.26 | 0.00 | 1.17 | 1.35 | 1.17 | 0.00 | 1.08 | 1.27 |
| Partner | 1.36 | 0.00 | 1.16 | 1.59 | 1.23 | 0.03 | 1.02 | 1.48 |
| Child | 1.49 | 0.00 | 1.23 | 1.80 | 1.55 | 0.00 | 1.24 | 1.94 |
| Parent | 2.38 | 0.00 | 2.04 | 2.78 | 1.52 | 0.00 | 1.26 | 1.82 |
| Parent-in-law | 1.83 | 0.00 | 1.38 | 2.43 | 1.08 | 0.63 | 0.78 | 1.51 |
| Other relative | 1.61 | 0.00 | 1.25 | 2.08 | 1.41 | 0.02 | 1.05 | 1.90 |
| Non-relative | 2.08 | 0.00 | 1.74 | 2.49 | 1.42 | 0.00 | 1.15 | 1.75 |

Adjusted model controls for outcome at baseline, care regimes, age, gender, living with partner, education, employment status, household wealth, longstanding limiting illness, and depression symptomatology.

**Supplementary Table 13.** Care status and volunteering frequency, 2019 results (adjustment for care in 2019) (N =15,546)

|  | **Adjusted** | | | |
| --- | --- | --- | --- | --- |
|  | AME | p-value | 95% CI | |
| **Non-care** |  |  |  |  |
| Reference | - | - | - | - |
| **Carer** |  |  |  |  |
| No volunteering | -0.05 | 0.00 | -0.06 | -0.03 |
| Every few months or less often | 0.00 | 0.41 | -0.01 | 0.01 |
| Almost every month | -0.00 | 0.32 | -0.01 | 0.00 |
| Twice a month or more | 0.05 | 0.00 | 0.03 | 0.06 |
| **Former carer** |  |  |  |  |
| No volunteering | -0.01 | 0.08 | -0.03 | 0.00 |
| Every few months or less often | -0.01 | 0.18 | -0.01 | 0.00 |
| Almost every month | -0.01 | 0.11 | -0.01 | 0.00 |
| Twice a month or more | 0.02 | 0.00 | 0.01 | 0.04 |

Adjusted model controls for outcome at baseline, *care status in 2019*, care regimes, age, gender, living with partner, education, employment status, household wealth, longstanding limiting illness, and depression symptomatology.

**Supplementary Table 14.** Care frequency and volunteering frequency, 2019 results (adjustment for care status in 2019) (N =15,546)

|  | **Adjusted** | | | |
| --- | --- | --- | --- | --- |
|  | AME | p-value | 905% CI | |
| **Non-care** |  |  |  |  |
| Reference | - | - | - | - |
| **Former carer** |  |  |  |  |
| No volunteering | -0.01 | 0.08 | -0.03 | 0.00 |
| Every few months or less often | -0.01 | 0.18 | -0.01 | 0.00 |
| Almost every month | -0.01 | 0.11 | -0.01 | 0.00 |
| Twice a month or more | 0.02 | 0.00 | 0.01 | 0.04 |
| **Daily care in household** |  |  |  |  |
| No volunteering | -0.03 | 0.04 | -0.06 | -0.00 |
| Every few months or less often | 0.01 | 0.41 | -0.01 | 0.02 |
| Almost every month | -0.01 | 0.12 | -0.02 | 0.00 |
| Twice a month or more | 0.03 | 0.01 | 0.01 | 0.06 |
| **Daily care outside household** |  |  |  |  |
| No volunteering | -0.04 | 0.03 | -0.08 | -0.00 |
| Every few months or less often | 0.00 | 0.89 | -0.02 | 0.02 |
| Almost every month | -0.00 | 0.64 | -0.02 | 0.01 |
| Twice a month or more | 0.04 | 0.00 | 0.01 | 0.08 |
| **Weekly care outside household** |  |  |  |  |
| No volunteering | -0.07 | 0.00 | -0.10 | -0.04 |
| Every few months or less often | 0.01 | 0.43 | -0.01 | 0.02 |
| Almost every month | -0.01 | 0.25 | -0.02 | 0.01 |
| Twice a month or more | 0.07 | 0.00 | 0.05 | 0.10 |
| **Monthly care** |  |  |  |  |
| No volunteering | -0.01 | 0.77 | -0.07 | 0.05 |
| Every few months or less often | -0.01 | 0.46 | -0.04 | 0.02 |
| Almost every month | 0.04 | 0.08 | -0.00 | 0.09 |
| Twice a month or more | -0.02 | 0.34 | -0.07 | 0.02 |

Adjusted model controls for outcome at baseline, *care status in 2019*, care regimes, age, gender, living with partner, education, employment status, household wealth, longstanding limiting illness, and depression symptomatology.

**Supplementary Table 15.** Relationship to care recipient and volunteering frequency, 2019 results (adjustment for care status in 2019) (N =15,546)

|  | **Adjusted** | | | |
| --- | --- | --- | --- | --- |
|  | AME | p-value | 905% CI | |
| **Non-care** |  |  |  |  |
| Reference | - | - | - | - |
| **Former carer** |  |  |  |  |
| No volunteering | -0.01 | 0.08 | -0.03 | 0.00 |
| Every few months or less often | -0.01 | 0.18 | -0.01 | 0.00 |
| Almost every month | -0.01 | 0.11 | -0.01 | 0.00 |
| Twice a month or more | 0.02 | 0.00 | 0.01 | 0.04 |
| **Partner** |  |  |  |  |
| No volunteering | -0.05 | 0.01 | -0.08 | -0.01 |
| Every few months or less often | -0.00 | 0.97 | -0.02 | 0.02 |
| Almost every month | -0.01 | 0.27 | -0.02 | 0.01 |
| Twice a month or more | 0.06 | 0.00 | 0.03 | 0.08 |
| **Child** |  |  |  |  |
| No volunteering | -0.04 | 0.05 | -0.09 | 0.00 |
| Every few months or less often | 0.04 | 0.01 | 0.01 | 0.07 |
| Almost every month | -0.01 | 0.16 | -0.03 | 0.01 |
| Twice a month or more | 0.02 | 0.34 | -0.02 | 0.05 |
| **Parent** |  |  |  |  |
| No volunteering | -0.02 | 0.24 | -0.05 | 0.01 |
| Every few months or less often | 0.01 | 0.16 | -0.01 | 0.03 |
| Almost every month | 0.00 | 0.96 | -0.02 | 0.02 |
| Twice a month or more | 0.00 | 0.72 | -0.02 | 0.03 |
| **Parent-in-law** |  |  |  |  |
| No volunteering | -0.02 | 0.46 | -0.08 | 0.03 |
| Every few months or less often | 0.00 | 0.77 | -0.03 | 0.04 |
| Almost every month | 0.01 | 0.56 | -0.02 | 0.04 |
| Twice a month or more | 0.01 | 0.74 | -0.04 | 0.05 |
| **Other relative** |  |  |  |  |
| No volunteering | 0.02 | 0.49 | -0.03 | 0.06 |
| Every few months or less often | -0.02 | 0.14 | -0.04 | 0.01 |
| Almost every month | -0.00 | 0.72 | -0.03 | 0.02 |
| Twice a month or more | 0.00 | 0.83 | -0.03 | 0.04 |
| **Non-relative** |  |  |  |  |
| No volunteering | -0.10 | 0.00 | -0.14 | -0.06 |
| Every few months or less often | 0.01 | 0.21 | -0.01 | 0.04 |
| Almost every month | -0.00 | 0.81 | -0.02 | 0.02 |
| Twice a month or more | 0.09 | 0.00 | 0.06 | 0.13 |

Adjusted model controls for outcome at baseline, *care status in 2019*, care regimes, age, gender, living with partner, education, employment status, household wealth, longstanding limiting illness, and depression symptomatology.

**Supplementary Table 16.** Care status and group membership (adjustment for care status in 2019) (N=14,800)

|  | Adjusted | | | |
| --- | --- | --- | --- | --- |
| Group membership | OR | p-value | 95% CI | |
| Non-carer (Reference) | - | - | - | - |
| Carer | 1.15 | 0.04 | 1.01 | 1.32 |
| Former carer | 1.00 | 0.93 | 0.90 | 1.12 |

Adjusted model controls for outcome at baseline, *care status in 2019*, care regimes, age, gender, living with partner, education, employment status, household wealth, longstanding limiting illness, and depression symptomatology.

**Supplementary Table 17.** Care frequency and group membership (adjustment for care status in 2019) (N=14,800)

|  | Adjusted | | | |
| --- | --- | --- | --- | --- |
| Group membership | OR | p-value | 95% CI | |
| Non-carer (Reference) | - | - | - | - |
| Former carer | 1.00 | 0.93 | 0.90 | 1.12 |
| Daily in household | 1.15 | 0.20 | 0.93 | 1.43 |
| Daily outside household | 1.11 | 0.45 | 0.84 | 1.46 |
| Weekly outside household | 1.19 | 0.13 | 0.95 | 1.48 |
| Monthly | 1.15 | 0.58 | 0.71 | 1.85 |

Adjusted model controls for outcome at baseline, *care status in 2019*, care regimes, age, gender, living with partner, education, employment status, household wealth, longstanding limiting illness, and depression symptomatology.

**Supplementary Table 18.** Relationship to care recipient and group membership (adjustment for care status in 2019) (N=14,800)

|  | Adjusted | | | |
| --- | --- | --- | --- | --- |
| Group membership | OR | p-value | 95% CI | |
| Non-carer (Reference) | - | - | - | - |
| Former carer | 1.01 | 0.93 | 0.90 | 1.12 |
| Partner | 1.06 | 0.62 | 0.83 | 1.36 |
| Child | 1.14 | 0.41 | 0.84 | 1.56 |
| Parent | 1.25 | 0.07 | 0.98 | 1.59 |
| Parent-in-law | 0.69 | 0.10 | 0.45 | 1.07 |
| Other relative | 1.00 | 1.00 | 0.68 | 1.48 |
| Non-relative | 1.30 | 0.05 | 1.00 | 1.69 |

Adjusted model controls for outcome at baseline, *care status in 2019*, care regimes, age, gender, living with partner, education, employment status, household wealth, longstanding limiting illness, and depression symptomatology.

| **SHARE** | **Wave 1**  **2004/05** | **Wave 2**  **2006/07** | **Wave 4**  **2011/12** | **Wave 5**  **2013** | **Wave 6**  **2015** | **Wave 8**  **2019/20** |
| --- | --- | --- | --- | --- | --- | --- |
| **Baseline***  Joins the study as non-carer | **✓** | **✓** | **✓** | **✓** |  |  |
| **Transition to care**  After their baseline observation, participants are followed at each consecutive wave to assess if they remain as carers or transition into care at some point between baseline to wave 6. |  | **✓** | **✓** | **✓** | **✓** |  |
| **Care status**  Non-carer, carer, or former carer depending on data patterns from baseline to wave 6. |  |  |  |  | **✓** |  |
| **Outcomes** |  |  |  |  | **✓** | **✓** |

*Due to the presence of refreshment samples participants have different baselines.

*Wave 3 and wave 7 are excluded due to SHARELIFE questionnaire.*

**Supplementary Figure 1.** Timeline for measurement of study variables in SHARE

| **ELSA** | **Wave 2**  **2004/05** | **Wave 3**  **2006/07** | **Wave 4**  **2008/09** | **Wave 5**  **2010/11** | **Wave 6**  **2012/13** | **Wave 7**  **2014/15** | **Wave 9**  **2018/19** |
| --- | --- | --- | --- | --- | --- | --- | --- |
| **Baseline***  Joins the study as non-carer | **✓** | **✓** | **✓** | **✓** | **✓** |  |  |
| **Transition to care**  After their baseline observation, participants are followed at each consecutive wave to assess if they remain as carers or transition into care at some point between baseline to wave 7. |  | **✓** | **✓** | **✓** | **✓** | **✓** |  |
| **Care status**  Non-carer, carer, or former carer depending on data patterns from baseline to wave 7. |  |  |  |  |  | **✓** |  |
| **Outcomes** |  |  |  |  |  | **✓** | **✓** |

*Due to the presence of refreshment samples participants have different baselines.

**Supplementary Figure 2.** Timeline for measurement of study variables in ELSA

**
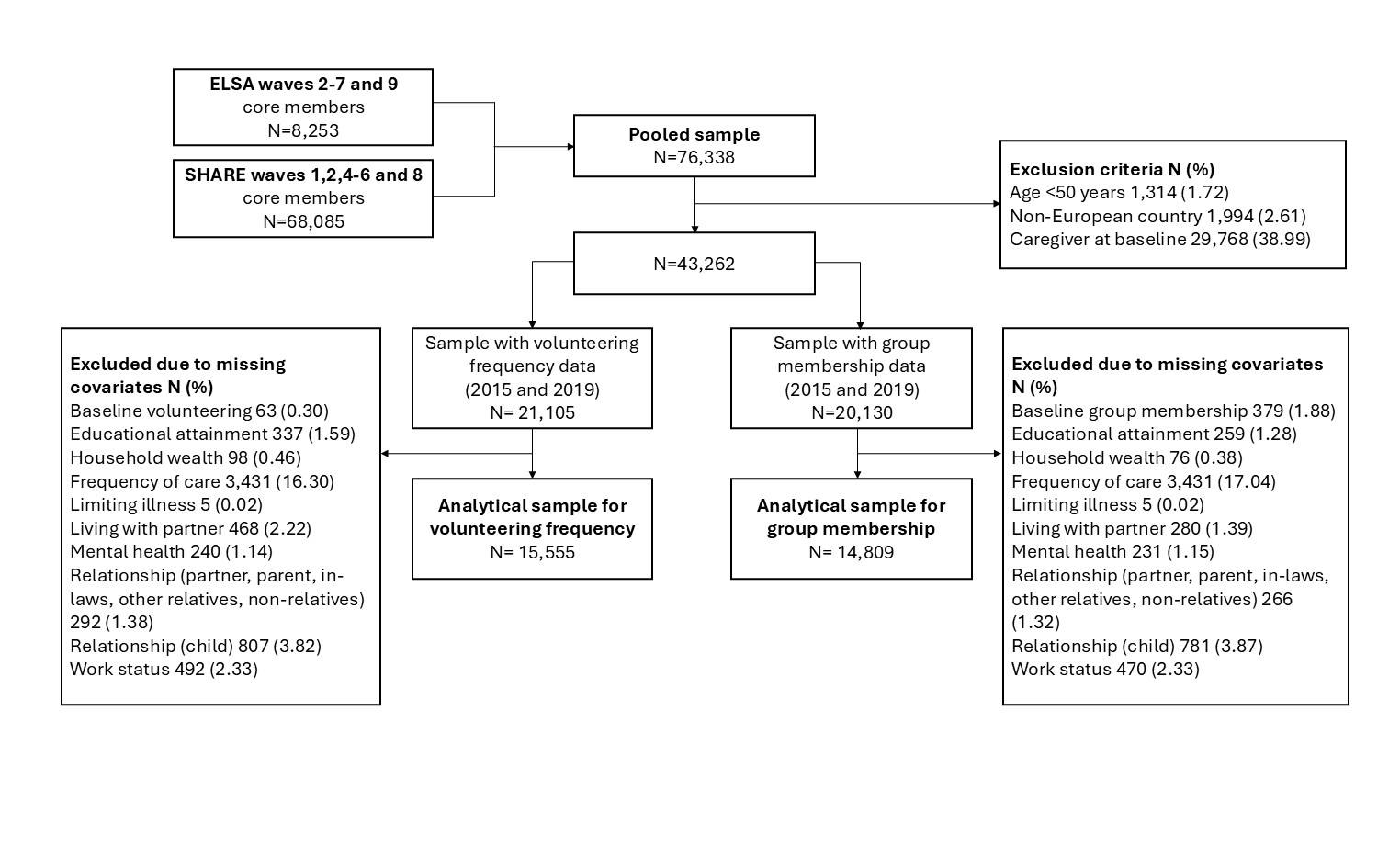
**

**Supplementary Figure 3.** Participant flowchart

**Supplementary Figure 4.** Timeline for measurement of study variables in ELSA

Three care patterns were identified from the carers group: (1) new carers (57.65%), representing individuals who transitioned into care in 2015 but had not provided care in any of the waves previously assessed; (2) continuous carers (30.92%), comprised of participants who provided care for at least two continuous waves or more; and (3) intermittent carers (10.71%), who transitioned to care at some point during the observation period, stopped for at least one wave, but then returned to care by 2015 (see Supplementary Figure 3 for illustration of data patterns in care categories). The remaining 0.71% of the sample was comprised of 10 participants who had missing data in at least one wave between their baseline and 2013, and thus, could not be classified into the three patterns described above.


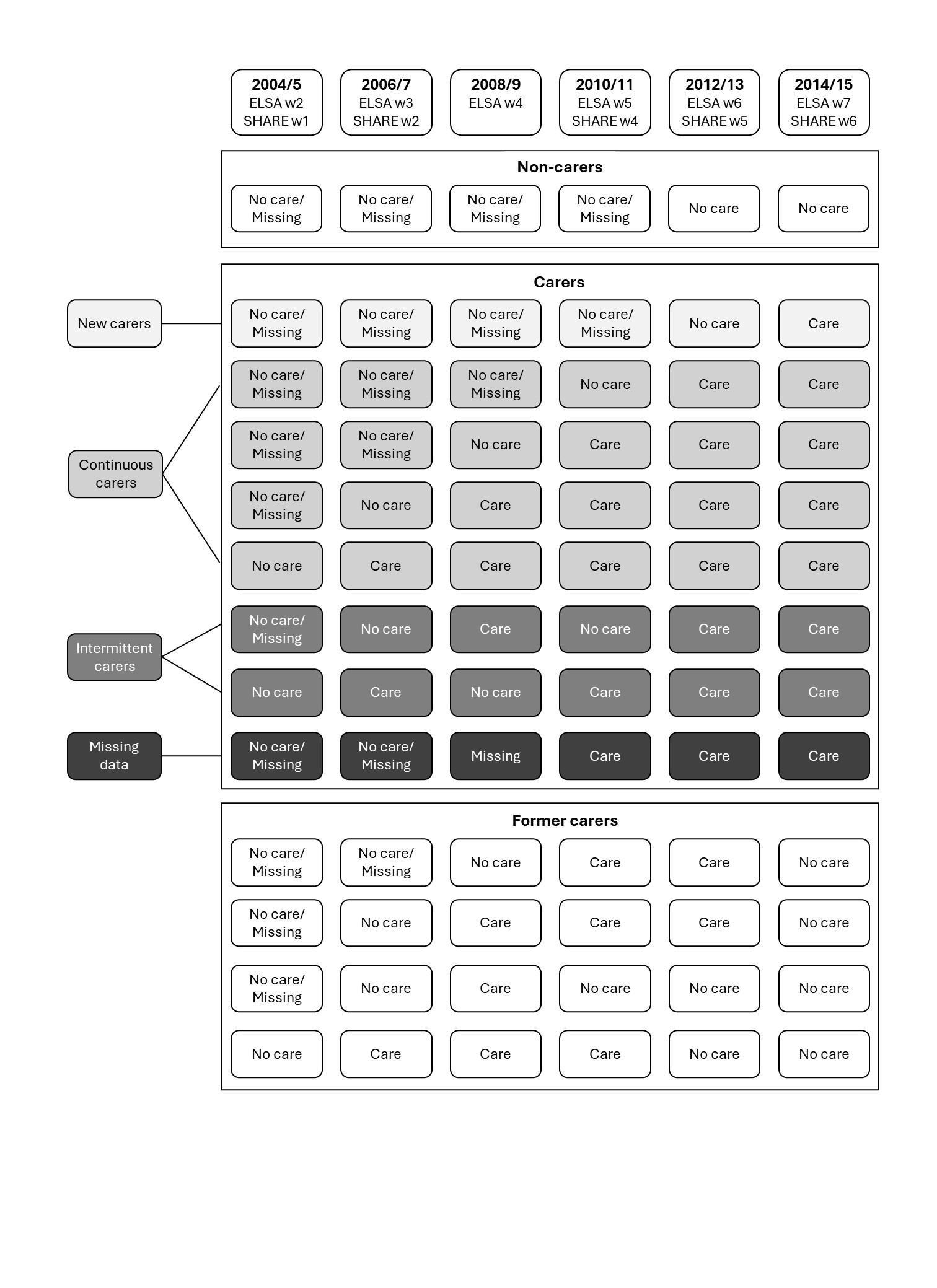
**Supplementary Figure 4**. Examples of missing and care variable patterns within each care status (non-carer, carer, former carer)

|  |
| --- |
|  |
|  |

**Supplementary Figure 5.** Care and volunteering frequency (2015) stratified by country care regimes

Non-carers set as reference category.


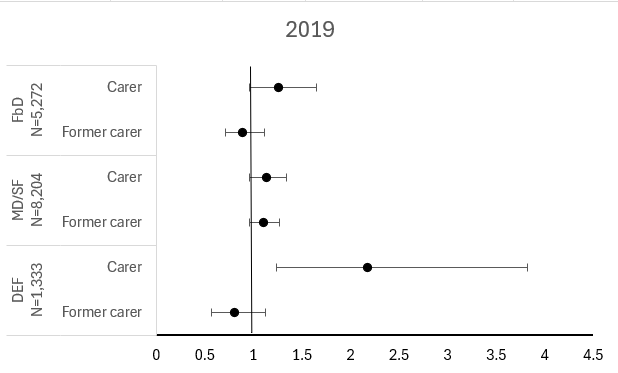


**Supplementary Figure 6.** Care and group membership (2019) stratified by country care regimes.

Non-carers set as reference category.
